# Inducible dCas12a system expands CRISPRi/a capabilities for functional genomics in malaria parasite *Plasmodium falciparum*

**DOI:** 10.1016/j.isci.2026.117704

**Published:** 2026-09-22

**Authors:** Amuza Byaruhanga Lucky, XiaoLian Li, George Kobakhidze, Jose Lulo Gonzales, Chengqi Wang, Liwang Cui, Gang Dong, Jun Miao

**Affiliations:** 1 Department of Internal Medicine, University of South Florida, 3720 Spectrum Blvd, Tampa, FL, USA; 2 Max Perutz Labs, Vienna Bio-center, Center for Medical Biochemistry, Medical University of Vienna, Dr. Bohr-Gasse 9/3, 1030 Vienna, Austria; 3 College of Engineering, Department of Medical Engineering, University of South Florida, 4202 E. Fowler Avenue, ENG 030, Tampa, FL, USA; 4 Center for Global Health and Infectious Diseases Research, College of Public Health, University of South Florida, 3720 Spectrum Blvd, Tampa, FL, USA

**Keywords:** human malaria, *Plasmodium falciparum*, CRISPR, dCas12a, gene regulation

## Abstract

We developed inducible dead Cas12a (dCas12a)-based CRISPR interference/activation (CRISPRi/a) systems for *Plasmodium falciparum*, expanding the gene regulation toolkit beyond dCas9. Our previous systems fused dCas9 to the catalytic domains of PfSir2a or PfGCN5 under DiCre/*loxP* regulon, enabling conditional gene regulation but remaining constrained by Cas9’s NGG protospacer adjacent motif (PAM) requirement. Unlike dCas9, dCas12a recognizes T-rich PAMs and processes a single transcript into multiple sgRNAs via its RNase activity, allowing multiplexed regulation of one or multiple genes. Functional validation targeting *EBA175* and *ATG18* demonstrated that single sgRNAs mediated CRISPRi/a’s active gene regulation, while multiplexed sgRNA arrays enhanced CRISPRi activities. Using this platform, we showed that conditional repression of the histone methyltransferase gene *PfSET7* impairs schizont egress and merozoite invasion, highlighting its critical role in parasite multiplication, consistent with its *Toxoplasma* homolog *TgADTM1*. Altogether, these results establish dCas12a-based CRISPRi/a as a versatile platform for conditional and multiplexed gene regulation in *P*. *falciparum*.

## Highlights

• Inducible dCas12a enables CRISPRi/a in *P*. *falciparum*
• dCas12a expands PAM availability and gRNA design space in *P*. *falciparum*
• gRNA arrays enhance repression but not activation of target genes
• PfSET7 repression impairs schizont egress and merozoite invasion

## Graphical abstract

**Figure:**
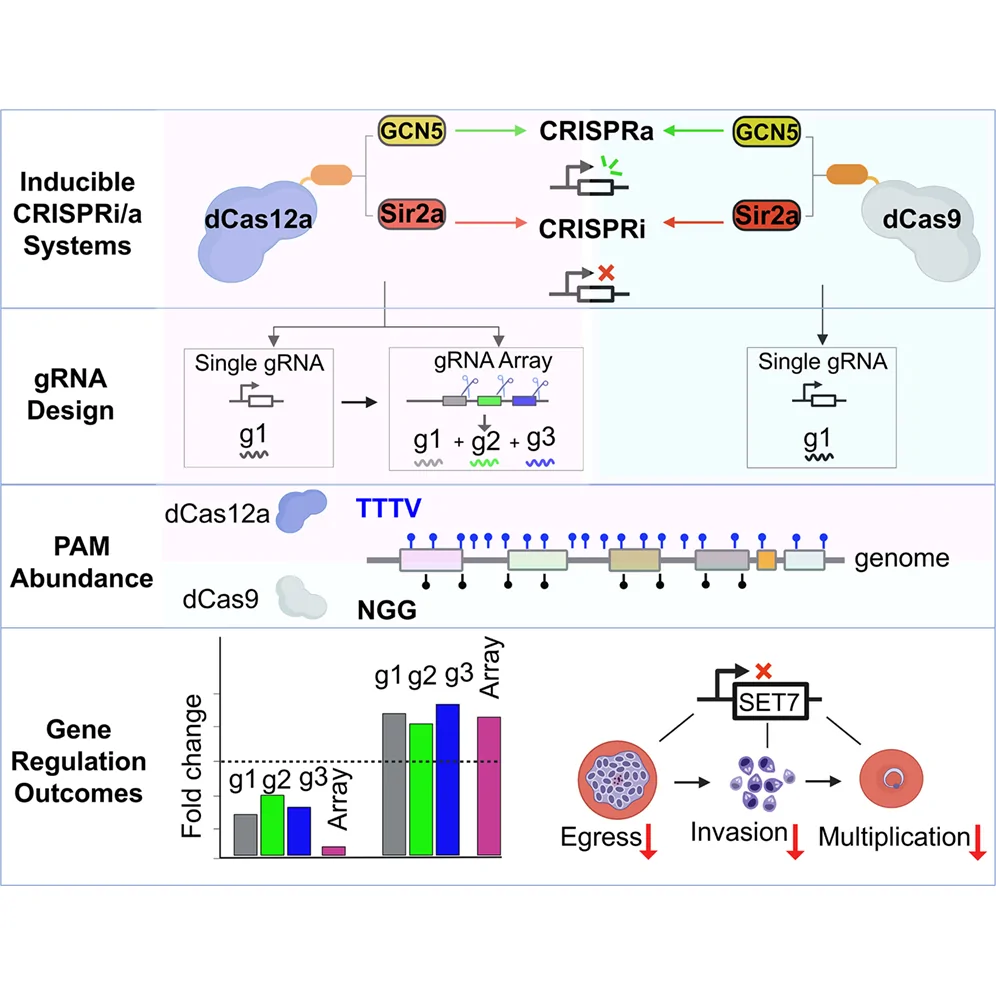

## Teaser

Microbial genomics; Molecular biology; Parasitology

## Introduction

Besides being successfully introduced into many species for gene manipulation, the CRISPR-Cas9 system has also been modified for various other genome engineering purposes, particularly for CRISPR interference and activation (CRISPRi/a). These approaches utilize a catalytically inactive Cas9 (dead Cas9 or dCas9) fused to regulatory factors to modulate target gene expression when dCas9 fusion proteins are guided to the promoter/enhancer regions by sgRNAs.^1^^,^^2^^,^^3^^,^^4^^,^^5^^,^^6^^,^^7^^,^^8^^,^^9^^,^^10^^,^^11^^,^^12^^,^^13^^,^^14^^,^^15^ For example, dCas9 fused to the Krüppel-associated box domain of Kox1 caused 15-fold repression of the target genes, whereas dCas9 fused to the p300 histone acetyltransferase (HAT) domain activated target genes.^6^^,^^10^ Compared with CRISPR-Cas9-mediated gene manipulation, CRISPRi/a systems offer the advantage of studying essential genes and non-coding RNAs, as they do not genetically modify DNA sequences.^15^^,^^16^^,^^17^

More than ten different CRISPR-Cas proteins have been repurposed for genome engineering besides Cas9. Among them, Cas12a (Cpf1) has drawn particular attention due to its three distinct features.^18^^,^^19^^,^^20^ First, Cas12a has RNase activity to process a CRISPR array, producing multiple gRNAs from a single transcript. Second, Cas12a associates only with the crRNA (no tracrRNA) for its endonuclease activity. Third, the protospacer adjacent motif (PAM) for Cas12a is T-rich (TTTV, V can be C, G, or A),^18^^,^^19^^,^^20^ which would greatly facilitate target site selection in AT-rich genomes such as that of the malaria parasite *Plasmodium falciparum*.^21^ Cas12a has been rapidly applied in genome engineering, both in multiplexed and inducible modules, for catalytically active Cas12a-based gene editing or catalytically inactive dCas12a-based regulation.^22^^,^^23^^,^^24^^,^^25^

Malaria still caused over half a million deaths (610,000) in 2024, primarily due to infection by the most virulent malaria parasite, *P*. *falciparum*.^26^ This parasite’s development inside the red blood cell is the clinically relevant stage for malaria pathology. Although the *P*. *falciparum* genome encodes over 5,000 genes, only about half of them have been functionally annotated.^27^ Therefore, additional tools are needed to decipher gene functions, given that the parasite has developed resistance to front-line antimalarials.^28^^,^^29^^,^^30^ The catalytically active CRISPR-Cas9 or Cas12a system has been adopted for gene disruption/insertion and point mutation in malaria parasites.^31^^,^^32^^,^^33^^,^^34^ CRISPRi was first adapted in *P*. *yoelii* by targeting dCas9 alone to the upstream region of *PyALBA4*, yielding 2– to 3 fold transcriptional repression.^35^ In contrast, dCas9 with a gRNA targeting the upstream region of *PfMT-A70* led to only ∼15%–40% reduction, indicating interference activity by dCas9 alone in *P*. *falciparum* was limited.^36^ Xiao et al. developed CRISPRi/a systems by fusing dCas9 to either PfSir2a, a class III histone deacetylase, or the HAT domain of PfGCN5 to repress or activate target genes by altering histone acetylation levels at their promoters.^37^ When targeted to the 5′ untranslated region (UTR) of *rh4* in Dd2 parasites, the dCas9-GCN5 significantly induced *rh4* expression, resulting in the switching of the parasites to sialic acid-independent invasion pathways. Conversely, when dCas9-Sir2a was targeted to the 5′UTR of *eba-175*, it repressed *eba-175* expression to ∼16% of that in 3D7 parasites, reducing the invasion rate to 30%. Furthermore, dCas9-Sir2a-mediated downregulation of the essential gene PfSET1 by 50% resulted in significant growth defects in the parasite.

To study gene function more precisely in *P*. *falciparum*, we recently converted the dCas9-based CRISPRi/a systems into inducible versions by incorporating a DiCre/*loxP* regulon. Continuous gene activation or repression may cause cell death or adaptation thus masking true phenotypes. Inducible regulation provides greater experimental control. These inducible systems could down- and up-regulate 8 selected genes at low, medium, and high expression levels in the asexual blood stage, including activation of *AP2-G*, a silent gene encoding a master regulator of gametocytogenesis, resulting in increased gametocyte production.^38^ Additionally, these systems also function in gametocytes.^38^ Furthermore, we have demonstrated that dCas9-based CRISPRi/a systems are robust and reproducible for forward genetic screening in *P*. *falciparum*, using a small-scale CRISPRi/a library targeting nine genes under competitive growth, drug (artemisinin and chloroquine) treatment, and stress (heat shock and starvation) conditions.^39^ Notably, dCas9-based CRISPRa/i achieves bidirectional gene regulation (activation and repression), which provides reciprocal evidence for gene essentiality/function.^38^^,^^39^

Cas12a offers two key advantages for its usage in *P*. *falciparum*: T-rich PAM recognition and the ability to process a single transcript into multiple gRNAs. Thus, in this study, we leveraged these features to generate dCas12a-based CRISPRi/a systems and evaluate their ability to manipulate gene expression in *P*. *falciparum*. Compared to the dCas9-based CRISPRa/i, dCas12a-based CRISPRa/i provided a more versatile platform for dissecting gene function in *P*. *falciparum*.

## Results

### High abundance of Cas12 PAM sites in the *P*. *falciparum* genome and promoters

To compare the distribution of PAM sites recognized by Cas9 and Cas12a in *P*. *falciparum*, we systematically scanned for Cas9 PAMs (NGG) and Cas12a PAMs (TTTV) in the genome of the widely used lab strain *P*. *falciparum* 3D7, which is derived from the NF54 isolate originating from Africa.^40^ Cas12a PAM sites are more than twice as abundant as Cas9 PAM sites in the genome (Figure 1A). Analysis of their genomic distribution showed that approximately 70% of Cas9 PAMs are within coding regions and ∼25% in intergenic regions. In contrast, over 40% of the Cas12a PAM sites are intergenic (Figure 1B). Further analysis of the PAMs in the available promoter regions, which were defined based on the annotated 5′ UTRs,^41^ indicates that Cas12a PAM sites are ∼5-fold more than Cas9 PAM sites in the promoters (Figure 1C; Table S1). After filtering out sgRNAs with predicted high off-target effects, the potential Cas12a-based sgRNAs for CRISPRa/i are ∼7-fold more than Cas9-based ones (Figure 1C; Table S2). Considering only gRNAs without predicted off-target effects in the 5′ UTRs, Cas12a identifies ∼8 gRNAs per gene compared to ∼3 for Cas9 (Tables S1 and S2). Using the *eba-175* gene as an example, Cas12a PAM sites are nearly 8-fold more frequent than Cas9 PAM sites in the annotated 804-bp 5′ UTR promoter, providing a substantially greater number of potential sgRNA target sites and, among them, three Cas12a sgRNAs but only one Cas9 sgRNA without potential off-target effects were deemed suitable for CRISPR-based gene manipulation (Figures 1D and S1). These findings indicate that Cas12a provides markedly broader target coverage, particularly within promoter regions, thereby facilitating more flexible and efficient sgRNA selection for CRISPR-based gene regulation in *P*. *falciparum*.

**Figure 1:**
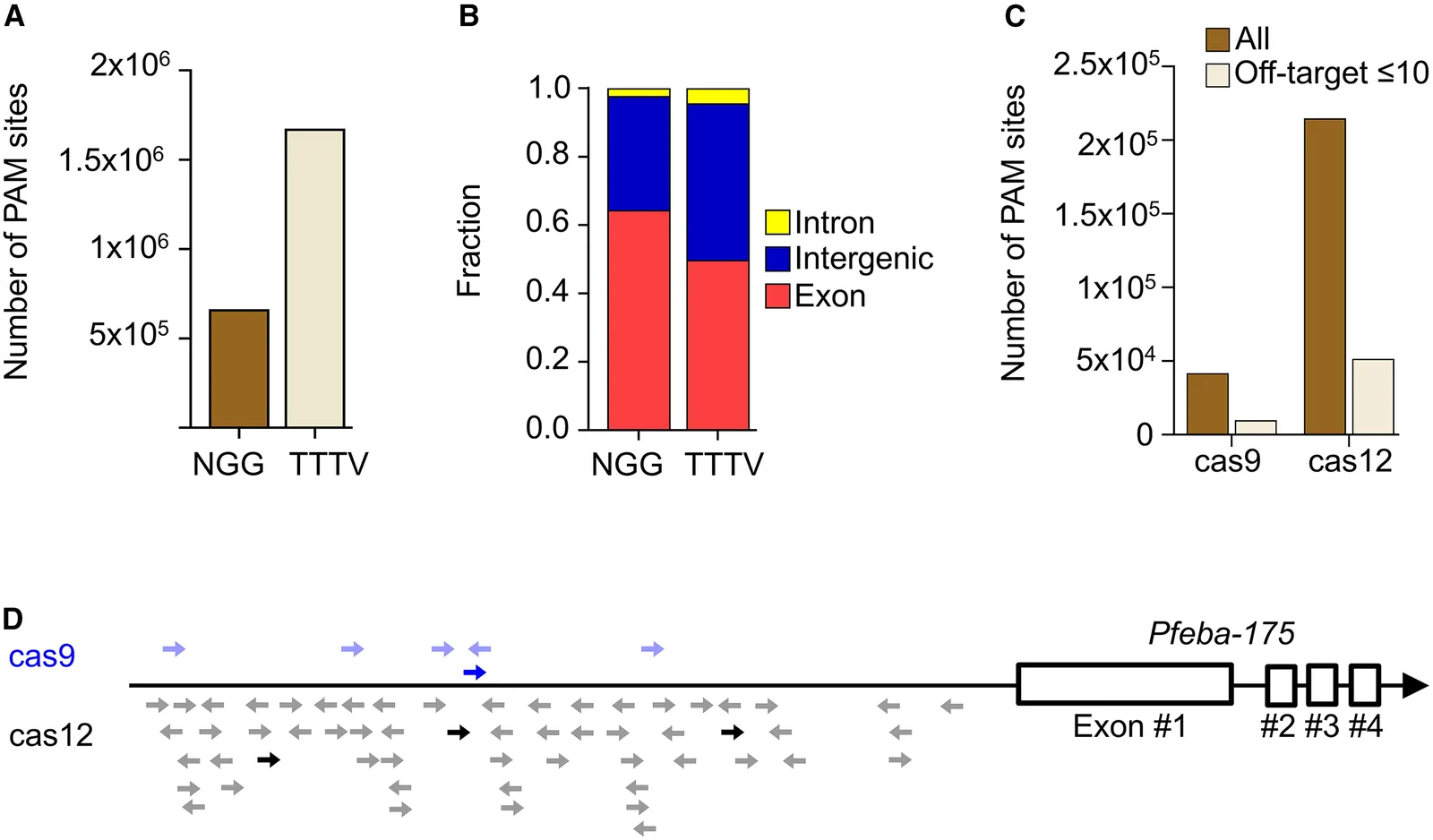
The PAM landscape and gRNA availability for dCa12a and dCas9-based CRISPRi/a systems in the *P*. *falciparum* (A) Genome-wide distribution of PAM sequences in the *P*. *falciparum* 3D7 genome. The TTTV PAM required by Cas12a is ∼2.5-fold more abundant than the NGG PAM required by Cas9, providing broader global gRNA design flexibility. (B) Genomic distribution of PAM sites across exons (red), intergenic regions (blue), and introns (yellow). (C) Genomic distribution of PAM sites across annotated 5′UTR promoter regions including all identified sites and filtered sites with low predicted off-target burden. Cas12a-based sites are substantially higher than Cas9. (D) Representative map of all sgRNAs upstream of the *eba-175* start codon (804 bp of the annotated 5′UTR). Blue and black arrows indicate Cas9 and Cas12a sgRNAs, respectively. Right and left pointing sgRNAs denote targeting of the non-template and template strands, respectively. Darker shaded arrows indicate sgRNAs with no predicted off-targets according to CRISPOR (see details in STAR Methods). The 4 boxes of the gene body denote exons. The higher density of Cas12a sgRNAs illustrates the increased design space and target versatility compared to Cas9.

### Generation of inducible dCas12a-based CRISPRi/a systems

We previously built inducible dCas9-based CRISPRi/a systems by inserting a green fluorescent protein (GFP) cassette between the constitutive *hsp86* promoter and the open reading frame (ORF) of dCas9 fusions to PfGCN5 or dCas9-PfSir2a, a HAT or deacetylase.^38^ In this design, the GFP stop codon and 3′ UTR prevent the expression of dCas9 fusion proteins, while the two *loxP* sites positioned just upstream of the GFP start codon and downstream of *hsp86* 3′ UTR terminator enable DiCre-mediated excision of *loxP*-flanked regions upon rapamycin (Rap) induction. This leads to the activation of the expression of the downstream dCas9 fusions. To build the same inducible CRISPRi/a systems for dCas12a, we replaced dCas9 with 3 × HA-tagged nuclease-inactive LbCas12a mutant from *Lachnospiraceae bacterium* ND2006^21^^,^^25^^,^^42^^,^^43^ (Figure 2A). These resulting dCas12a-based CRISPRi/a plasmids (GFP-dCas12a-Sir2a or the GFP-dCas12a-GCN5) were transfected into a DiCre-expressing *P*. *falciparum* 3D7 line.^44^ In this parasite background, functional Cre recombinase activity is induced only after dimerization of split Cre fragments is triggered by rapamycin treatment.^44^ Blasticidin (BSD)-resistant parasites were selected following transfection. Before Rap treatment, GFP was ubiquitously expressed in parasites (Figure 2B, top), whereas dCas12a-PfGCN5 or dCas12a-PfSir2A fusion proteins were undetectable by Western blot using the anti-FLAG and LbCas12a antibodies (Figures 2C and S2). 30 h after Rap induction (using 100 nM for 2 h) done in the ring stage (0–6 h post-invasion), GFP fluorescence in trophozoites disappeared, and dCas12a-fusion proteins became detectable (Figures 2B, bottom; 2C). These results support DiCre-mediated excision and activation of dCas12a-based CRISPRi/a constructs comparable to our previously established dCas9-based systems.^38^ Moreover, compared to 3D7 DiCre parasites, expression of dCas12a-fusion proteins did not noticeably affect parasite growth or development (Figure S3). Although performing additional reporter-based analyses could further strengthen characterization of inducible dCas12a-based CRISPRi/a activity, our results below demonstrate sgRNA-dependent activation and repression of target genes following induction.

**Figure 2:**
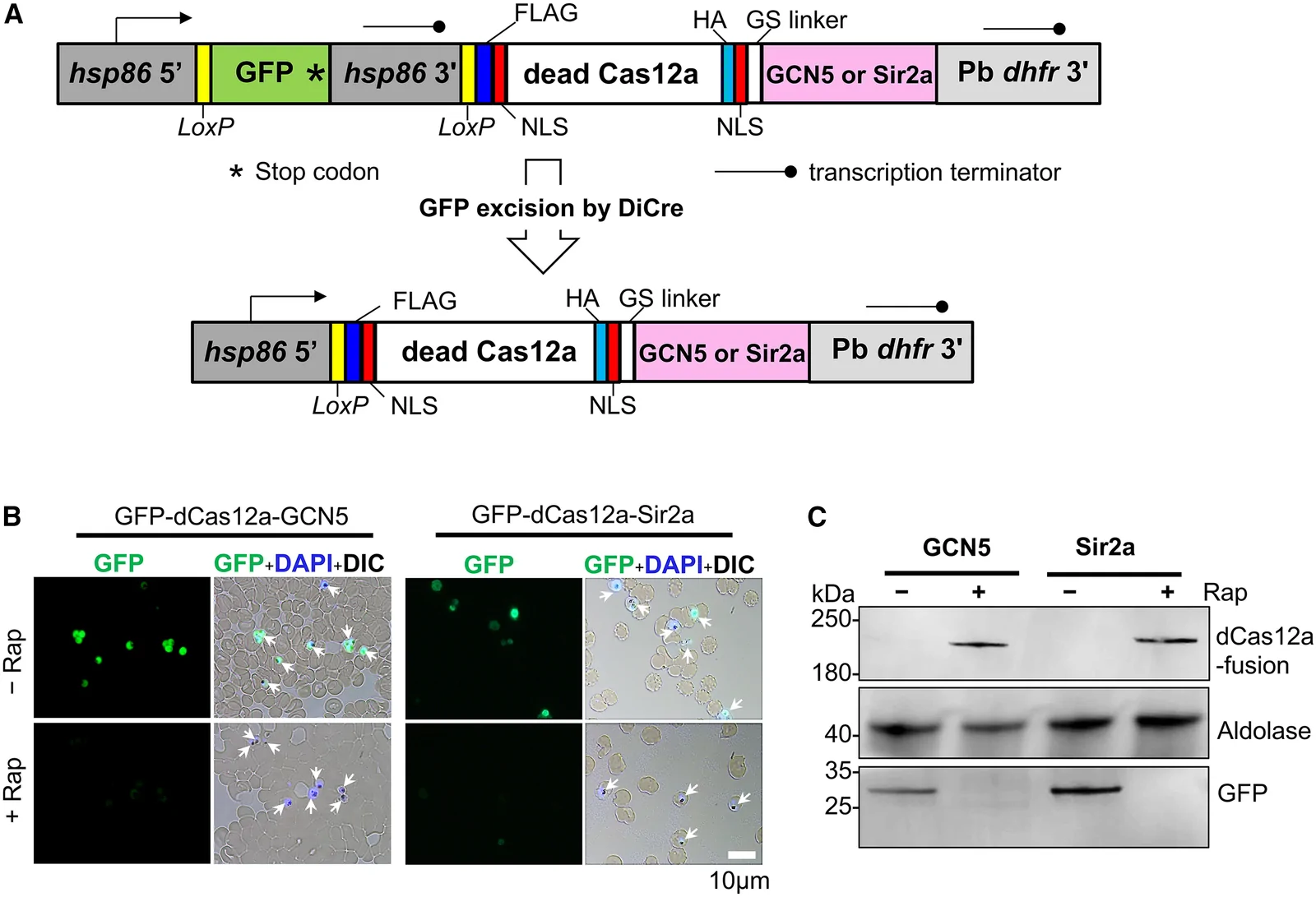
Generation of inducible dCas12a-GCN5/Sir2a systems using DiCre/*loxP* (A) A schematic illustrating the DiCre/*loxP* inducible CRISPR/dCas12a system. A GFP cassette with a stop codon followed by *hsp86* 3′ UTR flanked by *loxP* sites is inserted upstream of dCas12a-GCN5/Sir2a. Upon rapamycin (Rap) treatment, DiCre is activated, excising the *loxP* flanked GFP plus the *hsp86* 3′ UTR transcription terminator and enabling expression of dCas12a-GCN5/Sir2a. FLAG: FLAG tag; HA: HA tag; NLS: nuclear localizing signal; GS: glycine-serine linker, *Pb dhfr 3’*: *P*. *berghei* dhfr 3′ UTR transcription terminator. (B) Live-cell images showing GFP expression before (-Rap) and loss of GFP with concomitant appearance of dCas12a-fusion proteins 30 h after (+Rap) DiCre activation. White arrows indicate parasitized RBCs (scale bars, 10 μm). (C) Western blots confirm the switch from GFP to dCas12a-fusion protein expression following rapamycin treatment. PfAldolase was used as a loading control. Live imaging and western blots show loss of GFP expression and the induction of dCas12a-GCN5 and dCas12a-Sir2a protein expression following Rap treatment (at 0–6 h post-invasion, 2 h Rap exposure).

### dCas12a provides more versatile CRISPRi/a than dCas9 in *P*. *falciparum*

To measure dCas12a-based CRISPRi/a systems’ activities, we first established negative controls. In many published CRISPR-based studies, sgRNAs targeting non-essential genes or non-coding regions are generally selected to serve as negative controls.^45^ We used sgRNAs targeting *cg6* (*PF3D7_0709200*), a non-essential gene for asexual parasite growth that is frequently used for expression cassette insertion.^46^^,^^47^^,^^48^^,^^49^^,^^50^^,^^51^ Together with *mCherry*, which is absent from *P*. *falciparum*, were both included as our negative controls. Two sgRNAs targeting these loci (Figure S4A; Table S3) were introduced into GFP-dCas12a-Sir2a or -GCN5 parasite lines. As expected, parasite growth upon Rap induction was comparable to that without Rap induction or the parasites without sgRNA (Figure S3), confirming these controls did not affect growth (Figure S4B).

To compare the efficacy of dCas12a and dCas9, we first targeted *Pfatg18*. In our previous dCas9-based CRISPRi/a study, three sgRNAs located at 88, 757, and 1,409 bp upstream of *Pfatg18* repressed its expression to ∼30%, 50%, and 60%, respectively, or activated it by ∼2-, 2.3-, and 1.6-fold, respectively.^38^ Three dCas12a-based sgRNAs (g1, g2, and g3) located at 70, 580, and 1,396 bp upstream of *Pfatg18* start codon, respectively, corresponding to sites of dCas9-based sgRNAs, were selected for dCas12a-based CRISPRi/a (Figure 3A; Table S3). The plasmids carrying each sgRNA expression cassette were transfected into the GFP-dCas12a-Sir2a and the GFP-dCas12a-GCN5 parasite lines (Figure 3B top). Following Rap induction for 2 h in the early ring stage, total RNA was harvested at ∼40–46 h post-invasion (hpi) to measure the *Pfatg18* mRNA level by RT-qPCR. In the GFP-dCas12a-Sir2a line, *Pfatg18* expression was repressed to 10%–30% compared to control sgRNAs targeting *mCherry* (Figure 3C, *t* test, *p* < 0.01), causing marked growth defects (Figure 3D, ANOVA, *p* < 0.0001). Conversely, in the GFP-dCas12a-GCN5 line, g2 and g3 increased *Pfatg18* expression by ∼2.5- and 1.8-fold, respectively (Figure 3E, *t* test, *p* < 0.05), resulting in growth retardation (Figure 3F, ANOVA, *p* < 0.0001). g1 did not cause noticeable changes in *Pfatg18* expression (Figure 3E, *t* test, *p* > 0.05) and the corresponding parasites growth (Figure 3F, ANOVA, *p* > 0.05) in the GFP-dCas12a-GCN5 line.

**Figure 3:**
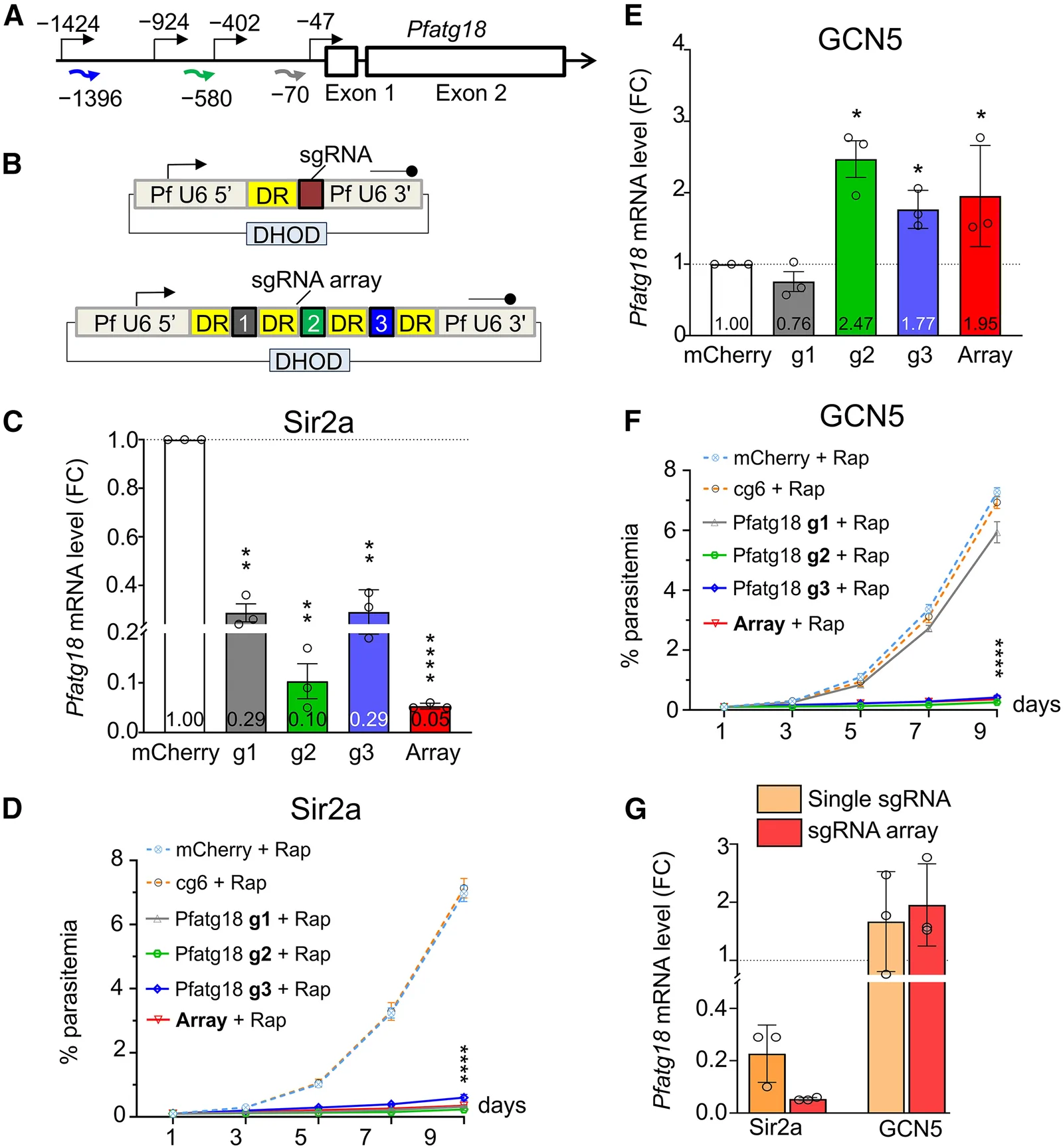
Inducible dCas12a-based CRISPRi/a regulates *Pfatg18* expression and parasite growth (A) Schematic showing the *Pfatg18* 5′UTR with transcription start sites (TSSs, arrows), the positions and orientation of 3 selected sgRNAs (wavy lines). (B) A schematic diagram of the gRNA expression plasmids (singlets and arrays, top and bottom, respectively) transfected into the dCas12a-Sir2a and the dCas12a-GCN5 parasite lines. The sgRNA array shows 3 sgRNAs (1, 2, 3) targeting a single gene (*Pfatg18* or *Pfeba-175*) expressed from a single cassette. Each sgRNA is flanked by direct repeat (DR) sequences and processed by dCas12 endonuclease activity. DHOD, dihydroorotate dehydrogenase drug cassette. (C) RT-qPCR analysis of *Pfatg18* transcript levels after Rap treatment in dCas12a-Sir2a parasites. Compared to control sgRNA targeting *mCherry*, g1, g2, g3, and the array repressed *Pfatg18* to ∼30%, 10%, 30%, and 5%, respectively. (D) Growth curves of dCas12a-Sir2a parasites showing *Pfatg18* growth retardation from day 3 compared to control sgRNAs targeting *mCherry* or *cg6* (broken lines). (E) RT-qPCR analysis of dCas12a-GCN5 lines showing activation of *Pfatg18* expression by sgRNA 2, 3, and the array, while sgRNA 1 had no effect. (F) Growth curves of dCas12a-GCN5 lines showing growth retardation in lines with elevated *Pfatg18* expression (sgRNA 2, 3, the array), whereas sgRNA1 parasites grew comparably to controls. (G) Multiple gRNAs enhanced the efficiency of dCas12a CRISPRi/a, as shown by improved regulation of *Pfatg18* expression compared to the average effect from individual sgRNAs in Figures 3C and 3E. Data represent mean ± SEM from 3 independent biological replicates. For each biological replicate, RT-qPCR was performed in technical triplicate for each gRNA, and the unpaired *t* tests were conducted (∗, ∗∗, ∗∗∗∗, *p* < 0.05, 0.01, 0.0001, respectively). Parasitemia was quantified from 3 independent biological replicates. Numbers in bars indicate fold-change (FC) values.

Similarly, we reported that dCas9-based CRISPRi with a gRNA located at 485 bp upstream of *Pfeba-175* repressed its expression to ∼16%.^37^ For dCas12a-based CRISPRi/a, three sgRNAs (g1, g2, and g3) were found at 230, 490, and 670 bp upstream of *Pfeba-175* near its transcriptional start sites (TSSs)^41^^,^^52^ (Figure 4A; Table S3). These gRNAs repressed *eba-175* expression to ∼23%, 15%, and 70%, respectively, in the GFP-dCas12a-Sir2a parasites (Figure 4B, *t* test, *p* < 0.01 and 0.001 for g1 and g2, respectively). Conversely, in the GFP-dCas12a-GCN5 parasites, *eba-175* expression was increased by ∼3.6-, 3.0-, and 2.8-fold, respectively (Figure 4C, *t* test, *p* < 0.05). Collectively, these data indicate that dCas12a-based CRISPRi/a systems are effective in mediating gene regulation and provide more sgRNA targeting options than Cas9-based systems. Given that gRNA sequence and secondary structure may influence CRISPRa/i activity, we additionally examined whether predicted gRNA folding stability correlated with activation or repression efficiency. Predicted minimum free energy (MFE; ΔG, kcal/mol) values for each sgRNA showed no strong correlation with either repression or activation (Figure S5; R^2^ = 0.1284 and 0.1120, respectively), suggesting that differences in predicted gRNA secondary structure are unlikely to be the primary determinant of the observed variability in gRNA performance.

**Figure 4:**
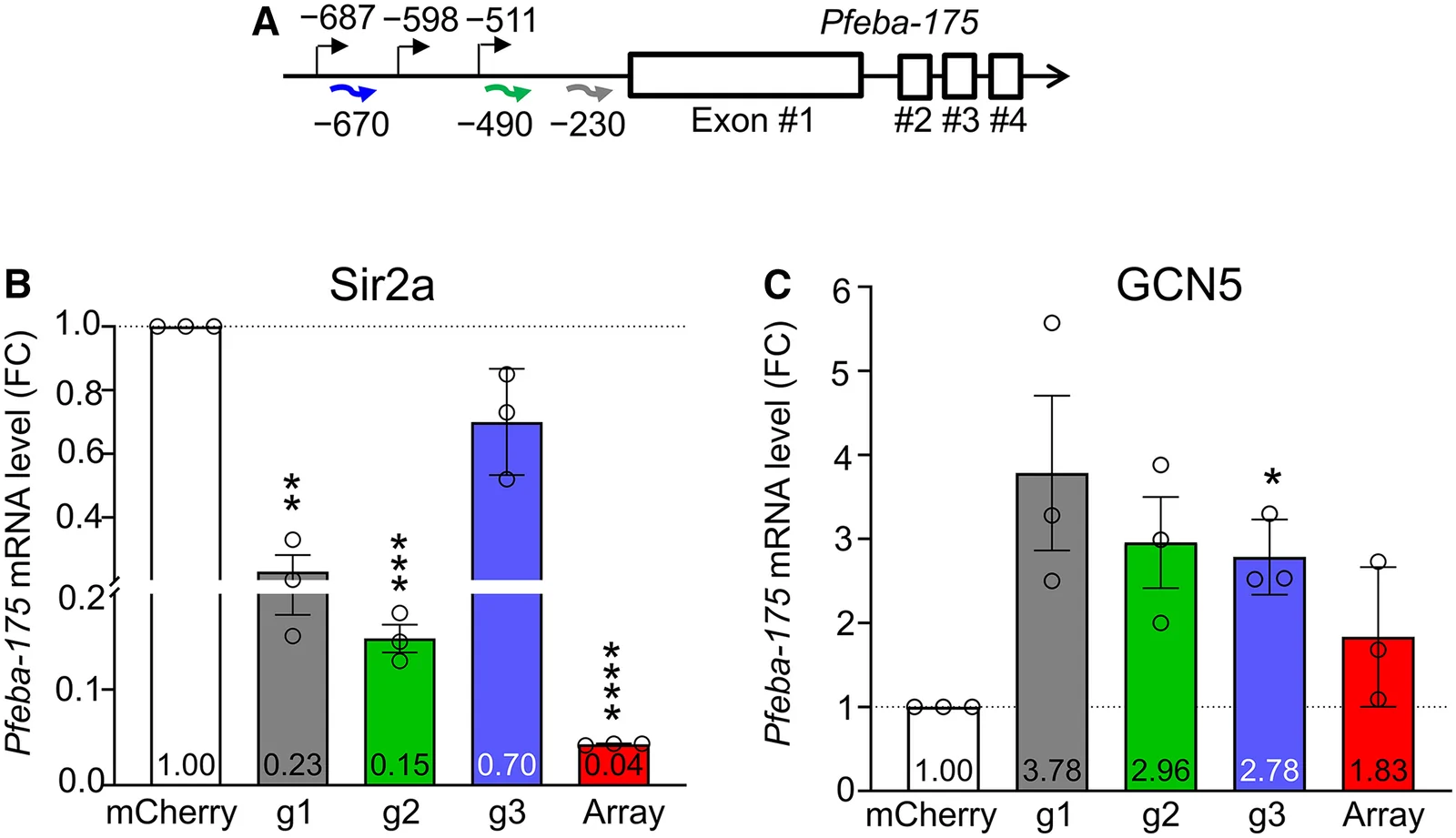
Interference and activation of *Pfeba-175* by inducible dCas12a systems (A) A schematic map showing the location of TSSs (arrows) with sgRNAs positions and orientation (wavy lines) in the 5′UTR of *Pfeba-175*. (B and C) *Pfeba-175* transcript level determined by RT-qPCR after repression in dCas12a-Sir2a (B) and activation in dCas12a-GCN5 (C) parasite lines. Three individual sgRNAs and an array were analyzed after Rap treatment compared to a control sgRNA targeting *mCherry*. Data represent mean ± SEM from three biological replicates; Numbers in bars indicate fold-change (FC) values. (Unpaired *t* test ∗, ∗∗, ∗∗∗, ∗∗∗∗: *p* < 0.05, 0.01, 0.001, 0.0001 respectively).

### Multiple gRNAs enhance dCas12a CRISPRi efficiency

To assess whether multiple gRNAs could enhance the activities in interference and activation for dCas12a-based CRISPRi/a, the three individual *Pfatg18-*targeting gRNAs were assembled as a single gRNA array in a single transcriptional cassette (Figures 3A and 3B bottom; Table S3). This resulting plasmid was introduced into GFP-dCas12a-Sir2a or -GCN5 parasite lines. Upon Rap induction, *Pfatg18* expression was reduced to <10% in GFP-dCas12a-Sir2a parasite line, whereas *Pfatg18* expression was increased ∼2-fold in GFP-dCas12a-GCN5 parasite line (Figures 3C and 3E). CRISPRi mediated by the array showed a non-significant trend toward stronger effects on *PfATG18* expression compared to single sgRNAs, while CRISPRa activity was comparable between array and single sgRNAs, with no statistically significant differences observed (Figure 3G). Parasites with either up- or down-regulated *Pfatg18* by CRISPRa/i guided by the array exhibited significant growth retardation (Figures 3D and 3F, ANOVA, *p* < 0.0001).

Similarly, a gRNA array consisting of three gRNAs targeting *Pfeba-175* reduced its expression to ∼4% in the GFP-dCas12a-Sir2a parasite line, a substantially stronger repression than that achieved with individual gRNAs (Figure 4B). In contrast, this array caused ∼2-fold increase of *Pfeba-175* expression in GFP-dCas12a-GCN5 parasite line, not higher than the individual sgRNA (Figure 4C). These results demonstrate that multiplexed gRNAs tend to enhance the efficiency and dynamic range of dCas12a-mediated CRISPRi regulation in *P*. *falciparum*.

### Dissection of the essentiality of PfSET7 by inducible dCas12a-based CRISPRi/a

To validate the utility of the inducible dCas12a-based CRISPRi/a system for studying essential genes, we targeted *Pfset7*, one of the nine SET domain-containing lysine methyltransferases (KMTs) in *P*. *falciparum*.^53^ Previous attempts to knockout this gene by double-crossover failed, suggesting that *Pfset7* is essential.^54^ Consistently, although *Pfset7* has a high mutagenesis index score (0.983) in a genome-wide piggyBac transposon mutagenesis screen, no insertions were observed within the locus.^27^ Phylogenetic analysis revealed that *Pfset7* is a homolog of *Toxoplasma gondii* apical complex KMT (TgAKMT),^55^ which localizes to the apical tip before egress and redistributes rapidly to the parasite body in response to egress-stimulating signals.^55^^,^^56^ Consistent with this localization pattern, *TgAKMT* deletion led to severe defects in parasite egress and invasion, resulting from the loss of motility.^56^

Three gRNAs (g1, g2, and g3) were designed to target *Pfset7* 5′UTR at 536, 1,289, and 1,985 bp upstream of the start codon, near a major (−2,083) and two minor (−1,237 and −86) transcription start sites (TSSs) based on the published TSS datasets^41^^,^^52^^,^^57^ (Figure 5A; Table S3). In GFP-dCas12a-Sir2a line, all three gRNAs significantly reduced *Pfset7* transcript levels, with g2 achieving the strongest (∼70%), g1 strong (∼60%), and g3 moderate (∼50%) repression (Figure 5B, *t* test, *p* < 0.001, 0.01, and 0.01, respectively). Western blot analysis with specific antibodies against PfSET7 confirmed the expression reductions for g1 and g2, except for g3, which exhibited a 1.4-fold expression increase in PfSET7 protein levels (Figures 5C, S6, and S7). Consistently, parasites with g1 and g2 but not g3 showed substantial growth inhibition (Figure 5D, ANOVA, *p* < 0.0001). Detailed analysis revealed egress defects in ∼39% and 64% of schizonts (Figures 5E and 5F *t* test, *p* < 0.001), while invasion efficiency of the purified merozoites decreased by ∼35% and 52% for g1 and g2, respectively, compared to control parasites (mCherry gRNA) (Figure 5G, *t* test, *p* < 0.001 and 0.0001). Taken together, these phenotypes indicate that PfSET7, like TgAKMT, is an essential gene and could play critical roles in parasite egress and invasion.

**Figure 5:**
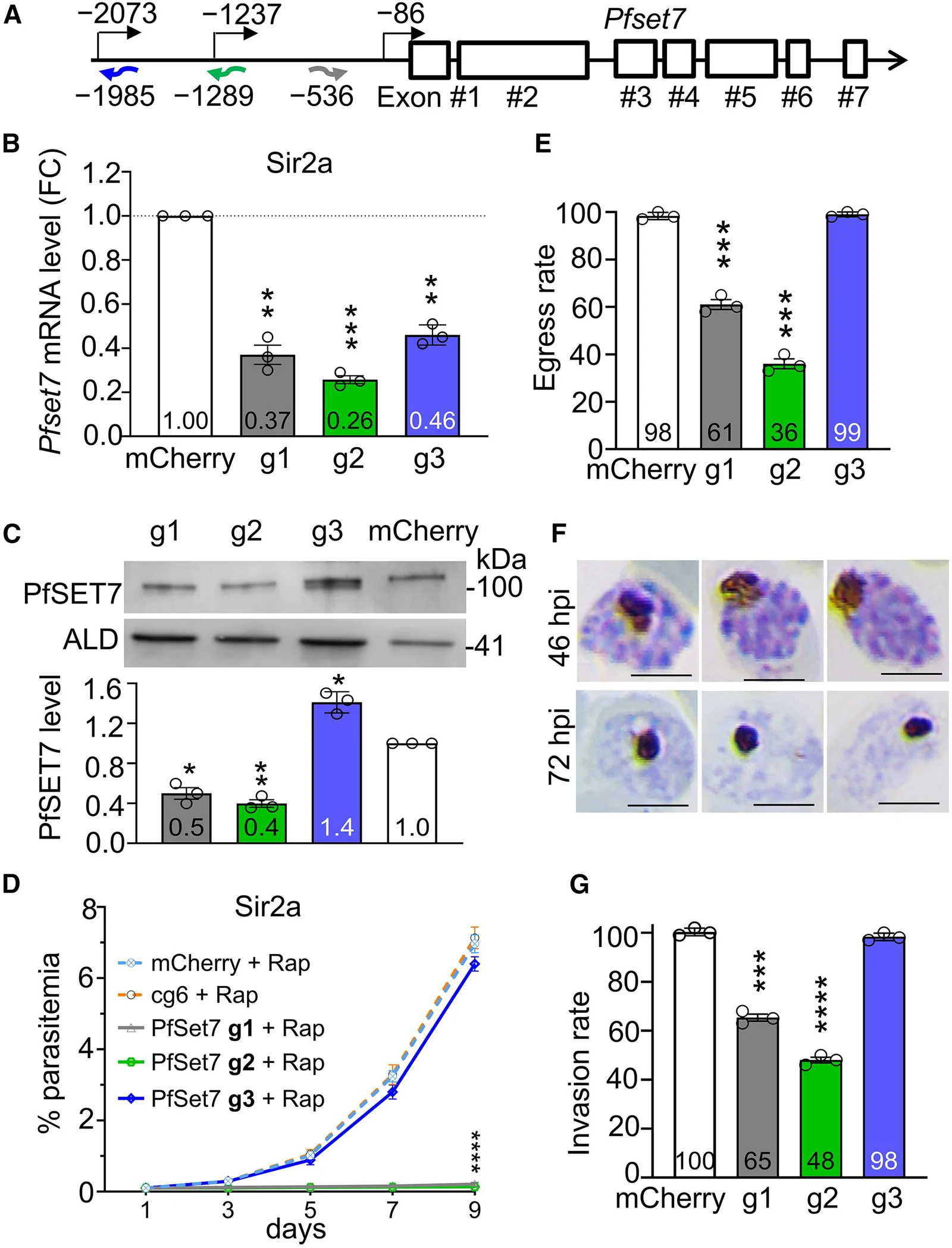
Alteration of *Pfset7* expression by dCas12a-CRISPRi/a impairs parasite egress and invasion (A) Schematic representation of the *Pfset7* locus showing transcription start sites (TSSs, arrows), positions and orientation of sgRNAs (wavy lines) in the 5′UTR. The gene blocks represent exons. (B) RT-qPCR in dCas12a-Sir2a parasites expressing 3 individual sgRNAs compared to the control sgRNA targeting *mCherry*. sgRNA 1 and 2 reduced transcript levels to ∼37%–26% of the control, while sgRNA3 had a moderate effect. (C) Western blot analysis of PfSET7 protein levels in parasites expressing individual sgRNAs relative to mCherry controls. PfAldolase (ALD) was used as a loading control. Quantification of relative PfSET7 protein levels is shown below. (D) Growth curves in dCas12a-Sir2a parasites expressing *Pfset7* sgRNAs or controls (*mCherry and cg6*) following Rap treatment. sgRNA1 and 2 reveal impaired parasite growth, whereas sgRNA3 and controls grow normally (ANOVA, *p* < 0.0001). (E) Egress assays performed in dCas12a-Sir2a parasites show reduced egress rates in Pfset7 sgRNA1 and 2 parasites, but not in sgRNA3 or controls. (F) Representative Giemsa-stained parasites expressing gRNA2 at 46 and 72 h post-invasion (hpi) are shown on the right (scale bars, 5 μm). (G) Invasion assays show decreased invasion efficiency in parasites expressing sgRNA1 and 2, consistent with reduced *Pfset7* expression. Data represent mean ± SEM from three biological replicates; Numbers in bars indicate fold-change (FC) values (*t* tests ∗, ∗∗, ∗∗∗, ∗∗∗∗: *p* < 0.05, 0.01, 0.001, 0.0001, respectively).

Conversely, the same three gRNAs led to ∼6.6-, 6.4-, and 3.7-fold increase in *Pfset7* expression in GFP-dCas12a-GCN5 parasite line, respectively (Figure S8A, *t* test, *p* < 0.01, 0.05, and 0.05). Interestingly, *Pfset7* overexpression also caused growth defects, with higher expression driven by g1 and g2 correlating with greater growth defects (Figure S8B, ANOVA, *p* < 0.0001). Together, these findings confirm that inducible dCas12a-based CRISPRi/a enables precise bidirectional (both up- and down-) regulation of essential genes and reveal that both depletion and overexpression of PfSET7 impair parasite viability.

## Discussion

In this study, we developed inducible dCas12a-based interference/activation (CRISPRi/a) systems for *P*. *falciparum*, expanding the functional genomic toolkit beyond dCas9-based approaches. By leveraging Cas12a’s preference for T-rich PAM sequences and its unique intrinsic RNase activity, which enables multiplex sgRNA processing, we achieved broader and more flexible targeting across the ∼80% AT-rich *P*. *falciparum* genome. This feature circumvents a major constraint of dCas9 systems that rely on NGG PAMs, which have limited target coverage.^18^^,^^19^ Notably, the inducible design of our dCas12a-based system provides temporal control over gene perturbation, mitigating parasite adaptation associated with constitutive modulation, an advantage we previously found for dCas9-based CRISPRi/a systems.^37^^,^^38^^,^^39^

Functional comparisons at the *Pfatg18* and *Pfeba-175* loci confirmed that dCas12a-based systems are more versatile than dCas9-based counterparts in both repression and activation. Furthermore, multiplexed gRNA arrays showed improved CRISPRi activities beyond that of individual sgRNAs, suggesting potential combinatorial contributions to gene expression perturbations and measurable parasite growth phenotypes. These results mirror findings from other eukaryotic models, where Cas12a-based multiplexing drives more potent and tunable gene regulation.^58^^,^^59^^,^^60^ In *P*. *falciparum*, where epistatic interactions and gene families are often co-regulated^61^^,^^62^^,^^63^ the ability to simultaneously modulate multiple targets or sites within a promoter presents a powerful approach for dissecting complex regulatory networks.

Using this system, we confirmed the essentiality of *Pfset7* and its potential function. Repression of *Pfset7* by CRISPRi impaired schizont egress and merozoite invasion, while upregulation of PfSET7 by CRISPRa led to parasite growth retardation. These results parallel those observed for the *Toxoplasma* homolog TgAKMT, underscoring conserved roles in parasite egress and motility.^55^^,^^56^ Together with results from *Pfatg18* perturbation, our data demonstrate that the inducible dCas12a-based CRISPRi/a platform provides a new tool for functional study of essential genes in *P*. *falciparum*. Unlike knockout approaches that yield only binary outcomes, the inducible system enables more nuanced analysis of essential gene functions that are inaccessible to direct knockout approaches due to lethality upon gene disruption.

Our recent report showed that activation of *Pfatg18* by dCas9-based CRISPRa led to growth defects.^38^ These results were recapitulated by using dCas12-based CRISPRa in this study (Figures 3E and 3F). *Pfatg18* is an important gene in the autophagy pathway.^64^^,^^65^^,^^66^ Inducing overexpression of *Pfatg18* may disrupt autophagy homeostasis, leading to growth impairment. Likewise, growth defects occurred upon activation of *Pfset7* by dCas12-based CRISPRa (Figure S8). Pfset7 encodes a methyltransferase.^53^^,^^54^ Pfset7 overexpression may lead to over-methylation of SET7 target proteins, which may disrupt essential cellular processes and affect parasite growth.

In bacteria and yeast, sgRNA orientation (template or non-template strand) has a profound impact on the dCas9-mediated repression of transcription elongation targeting gene coding regions.^67^^,^^68^^,^^69^ In contrast, both orientations of sgRNAs resulted in similar dCas9 activity in repressing transcription initiation when targeting 5′ UTR regions. All gRNAs in this study target the template strand at the 5′ UTRs of selected genes, except for two gRNAs (g2 and g3) targeting the non-template strand of *Pfset7* 5′ UTR (Figure 5A). Notably, g1 showed comparable activity to g2, which targets the non-template strand at *Pfset7* 5′ UTR.

In perspective, several attributes make this system particularly well-suited for large-scale functional genomics. First, the high density of TTTV PAMs in the *P*. *falciparum* genome ensures expanded targeting than dCas9, particularly in non-coding regulatory regions. Second, the ability of dCas12a to process multiple gRNAs from a single transcript reduces construct complexity for pooled screening, an approach already proven powerful in other eukaryotes.^59^^,^^70^^,^^71^ Third, like dCas9-based CRISPRa/i in *P*. *falciparum*,^39^ inducibility and bidirectional modulation of dCas12a-based CRISPRa/i will also enable dynamic phenotyping and uncover conditional gene requirements under drug pressure or environmental stress, enhancing the discovery of context-dependent essential genes.

### Limitations of the study

Despite these advantages, limitations remain. The degree of transcriptional modulation varies depending on sgRNA position relative to TSSs and local chromatin state, requiring empirical sgRNA optimization.^16^^,^^72^ Not all sgRNAs (1 of 9 sgRNAs for CRISPRa and 2 of 9 sgRNAs for CRISPRi) we used in this study showed substantial impacts on target genes’ expression. Since Cas12a expands PAM availability in promoters, this issue can be mitigated by using more sgRNAs (array) per target gene, as in other eukaryotes.^16^^,^^45^ Our data indicate that an array combined with CRISPRi enhanced repression, whereas an array coupled with CRISPRa resulted in activation of target genes but did not show enhanced effects compared to the effects from individual sgRNA, probably because activation by histone acetylation via CRISPRa is limited, especially when local histone acetylation is nearly saturated. Additionally, as with other CRISPR-based tools, confirmatory validation is needed to rule out potential off-target effects in *P*. *falciparum*. In this study, validation for *Pfset7* knockdown by CRISPRi with g3 is especially necessary because RT-PCR showed g3 led to ∼50% reduction in mRNA, but PfSET7 protein level was not reduced. The reason for this discrepancy could be a compensatory increase in PfSET7 translation in response to weak transcriptional perturbation. Given that the sgRNAs were selected using stringent off-target filtering, off-target effects are unlikely to account for this discrepancy, although they cannot be entirely ruled out.

In conclusion, we establish the inducible dCas12a-based CRISPRi/a system as a versatile and scalable tool for functional genomics in malaria parasites. Its inducibility and multiplexing capacity make it uniquely suited for dissecting essential genes and regulatory networks. This platform facilitates forward genetic screens and systematic identification of crucial pathways such as those underlying parasite development or invasion, accelerating the search for vulnerabilities and novel therapeutic targets.

## Resource availability

### Lead contact

Requests for further information and resources should be directed to and will be fulfilled by the lead contact, Jun Miao (jmiao1@usf.edu).

### Materials availability

Plasmids generated in this study have been deposited to (Addgene, pUF-dCas12-BSD-GCN5 # 260379 and pUF-dCas12-BSD-Sir2a # 260380). This study generated two parasite lines expressing CRISPR interference (DiCre/loxP-dCas12-BSD-Sir2a) and activation (DiCre/loxP-dCas12-BSD-GCN5). These reagents are available upon request.

### Data and code availability

• All data reported in this study will be shared by the lead contact upon request.
• This study does not report original code.
• Any additional information required to reanalyze the data reported in this study is available from the lead contact upon request.

## Acknowledgments

This study was funded by the 10.13039/100000060National Institute of Allergy and Infectious Diseases, National Institutes of Health (1R21AI149202 to J.M.), and the startup fund from 10.13039/100011280Morsani College of Medicine to J.M. We want to thank Dr. Michael Blackman for providing a *P*. *falciparum* clone engineered to express DiCre constitutively and Dr. Tobias Spielmann for providing the anti-PfAldolase antibodies.

## Author contributions

J.M., A.B.L., and L.C. conceptualized the study. L.C. and G.D. provided resources. A.B.L., X.L., G.K., and J.L.G. developed the methodology and performed the investigations. C.W. did the formal analysis. A.B.L. and J.M. drafted the manuscript. A.B.L., J.M., and L.C. reviewed and edited. J.M. supervised the study.

## Declaration of interests

We declare no conflicts of interest.

## STAR★Methods

### Key resources table

**Table:** 

REAGENT or RESOURCE	SOURCE	IDENTIFIER
**Antibodies**		

GFP Antibody	Novus Biologicals	NB100-1770SS; RRID:AB_10128178
FLAG Antibody	Abcam	ab1162; RRID:AB_298215
Anti-LbCas12a antibody	Sigma-Aldrich	SAB4200777-100UL; RRID:AB_2889872
Pf Aldolase	Dr. Tobias Spielmann	N/A
Donkey Anti-Goat IgG H&L (HRP)	Abcam	ab97110; RRID:AB_10679463
Goat Anti-Rabbit IgG Antibody, (H + L) HRP conjugate	Millipore sigma	AP307P; RRID:AB_92641
Pf SET7 antibody	Antibody developed in the authors’ lab	N/A

**Bacterial and virus strains**		

XL10-Gold Ultracompetent Cells	Agilent Technologies	200314

**Biological samples**		

DiCre/loxP-dCas12-BSD-GCN5	Parasite line generated in the authors’ lab	N/A
DiCre/loxP-dCas12-BSD-Sir2a	Parasite line generated in the authors’ lab	N/A

**Experimental models: Cell lines**		

Plasmodium falciparum 3D7A strain	BEI resources	MRA-151

**Oligonucleotides**		

Primer information (Table S3)	IDT DNA	N/A

**Recombinant DNA**		

pUF-dCas12-BSD-GCN5	This vector was modified in the authors’ laboratory	Addgene, # 260379
pUF-dCas12-BSD-Sir2a	This vector was modified in the authors’ laboratory	Addgene, # 260380

**Software and algorithms**		

SnapGene	https://www.snapgene.com/	8.2.2
CRISPOR	https://crispor.gi.ucsc.edu/ University of California at Santa Cruz	Version 5.2
CHOPCHOP	https://chopchop.cbu.uib.no/ University of Bergen	Version 3

### Experimental model and study participant details

This study used *Plasmodium falciparum* 3D7 strain. See the method details section for more information.

### Method details

#### Molecular cloning

To generate the inducible dCas12a-CRISPR i/a systems, the dCas9-GCN5 and dCas9-Sir2a expression cassettes^37^ were excised via *PacI* and *Xhol* and replaced by dCas12a-GCN5 and dCas12a-Sir2a expression cassettes. The dCas12a-GCN5 and dCas12a-Sir2a expression cassettes were assembled using a 2-fragment In-Fusion cloning system. The first fragment, dCas12a ORF, was PCR amplified from the WN10151 plasmid (Addgene # 80441) using primers dcas12.inf_F and dcas12.inf_R (Table S3). The second fragment, consisting of either the GCN5 or Sir2a domains,^37^ was PCR amplified with primer pairs HAT.infF/ HAT.infR or HDAC.infF/ HDAC.infR, respectively (Table S3). The two fragments were cloned into the *PacI* /*Xhol* digested backbone to generate the final expression vectors pUF-dCas12-BSD-GCN5 (# 260379, Addgene) and pUF-dCas12-BSD-Sir2a (# 260380, Addgene) (Figure S9).

For the gRNA-expressing vector, the AsCpf1 expression cassette was removed from the pAIO-AsCpf1 plasmid^33^ (gift from Josh Beck) by restriction digestion via *Smal* and *Sphl*. The resulting backbone left behind, containing the gRNA and DSM1 drug-selectable cassettes, was blunted and self-ligated. Individual gRNAs (target gene or decoy) and multiple gRNAs (arrays) were then cloned into the *AflII* site of this gRNA-expressing vector.

#### Selection and design of Cas12a-based gRNAs

Potential CRISPR target sites within *P*. *falciparum* genome were identified by scanning genomic sequences for canonical PAM motifs corresponding to SpCas9 (NGG) and Cas12a/Cpf1 (TTTV). For SpCas9 analysis, 20-bp guide sequences adjacent to NGG PAMs were extracted from both forward and reverse strands. For Cas12a analysis, 23-bp guide sequences associated with TTTV PAMs were identified similarly from both strands. Genomic coordinates, strand orientation, PAM sequences, guide sequences, and full target sequences were recorded for each candidate target site. Guide sequences were subsequently subjected to genome-wide off-target analysis using CRISPOR (v4.0)^73^^,^^74^ against the *P*. *falciparum* 3D7 reference genome (PlasmoDB v3.1) with PAM settings specific for Cas9 (NGG) and Cas12a (TTTV). CRISPOR was used to assess guide specificity and predict off-target burden. Guides with predicted off-target counts ≤10 were considered acceptable guide RNA candidates. The total number of filtered Cas9 and Cas12a targetable sites was subsequently summarized across genomic regions (Tables S1 and S2). For cloning and expression, gRNAs targeting the 5′ UTRs of selected genes were designed. gRNAs with high on-target scores (>50) and no predicted off-target sites with zero mismatches across the genome were prioritized for further analysis. Selected gRNAs were further cross validated using another prediction tool, CHOPCHOP v3.^75^ Two *PfATG18* gRNAs, all three *PfSET7* gRNAs, and one *Pfeba175* gRNA were consistently predicted by both tools. To evaluate whether selected gRNAs may form secondary structures that can contribute to variability in CRISPRa/i efficiency, predicted MFE values for individual gRNAs were calculated by RNA secondary structure prediction analysis using SnapGene 8.2.2 software. The predicted MFE values were subsequently compared with RT-qPCR repression or activation fold changes using linear regression analysis. Selected gRNA sequences were synthesized as single-stranded oligonucleotides, hybridized and cloned into the gRNA expression cassette using infusion cloning. For gRNA arrays, gRNA sequences were assembled as an array with intervening direct repeat (DR) sequences recognized by Cas12a endonuclease for crRNA processing. All constructs were verified by whole plasmid sequencing before transfection into *P*. *falciparum* parasites.

#### Parasite culture and transfection

*P*. *falciparum* clone constitutively expressing DiCre^44^ was kindly provided by Dr. Michael Blackman. Parasites were cultured in human red blood cells (RBCs) and cultured at 37°C in a gas mixture of 5% CO_2_, 3% O_2,_ and 92% N_2_ as previously described.^76^ Parasite transfection was performed using the RBC loading method.^77^ For the inducible dCas12a-GCN5 or -Sir2a system, the DiCre/*loxP*-based plasmids, pUF-dCas12-BSD-GCN5 and pUF-dCas12-BSD-Sir2a, were transfected into the DiCre-expressing parasite line, and resistant parasites were selected with BSD at 2.5 μg/ml.

#### Inducible expression of dCas12a-fusion proteins in the DiCre/loxP system

Purified schizonts collected on a 65/35% Percoll gradient were mixed with fresh RBCs for 4h to obtain tightly synchronized ring stage parasites. Rapamycin (100 nM) was added to the synchronized rings for 2 h, and parasites were collected 30 h after treatment. To confirm the expression of GFP and the dCas12a fusion proteins in the parasites before and after DiCre induction, GFP fluorescence signals in live parasites were captured using a Nikon Eclipse Ni epifluorescence microscope after the nuclei were stained by DAPI at 0.5μg/ml (Invitrogen). Western blot was done with goat anti-GFP (1:2000, Novus Biologicals), anti-FLAG (1:2000, Abcam), anti-LbCas12a (1:500, Sigma-Aldrich), and rabbit anti-PfAldolase (1:2000, kindly provided by Dr. Tobias Spielmann) antibodies as primary antibodies, followed by HRP-conjugated donkey anti-goat IgG and goat anti-rabbit IgG (Sigma) as secondary antibodies.

#### RNA isolation and reverse transcriptase-quantitative PCR (RT-qPCR)

Parasites were released from infected RBCs with 0.1% (v/v) saponin, and total RNA was extracted using the Quick-RNA MiniPrep kit (Zymo Research). cDNA synthesis was performed using SuperScript IV RT (Invitrogen), and qPCR with FastStart Universal SYBR Green Master Mix (Roche). Gene expression in the rapamycin-treated parasites was normalized to *seryl-tRNA synthetase* (PF3D7_0717700) using the 2^-ΔΔCt^ method^78^ and compared to control sgRNAs targeting *mCherry* or *cg6*. All experiments were independent biological replicates. Within each replicate, expression levels in parasites expressing sgRNA targeting mCherry were used as the control and set to 1.0, and test values were normalized to this replicate-specific control. Statistical analyses were performed on the then normalized fold-change values across biological replicates. Primer sequences are listed in Table S3. The data represent three biological replicates.

#### Phenotype analysis

The growth phenotypes of gRNA-expressing parasite lines were assessed in triplicate. Cultures were tightly synchronized and initiated at 0.5% rings, followed by rapamycin treatment. Parasitemia was monitored daily by Giemsa staining and phenotypic analysis was conducted for *Pfset7* knockdown lines. Tightly synchronized rings were cultured to mature schizonts. For egress assays, egress was analyzed by measuring the egress rate in the mature schizonts. Briefly, highly synchronized parasites at the mature schizont stage (∼46–48 h post-invasion) were purified by a 40%/70% Percoll-sorbitol gradient.^77^ The purified schizonts were put back into culture by adding new red blood cells and culture medium to allow schizont rupture and re-invasion, and the egress events were observed under live microscopy for at least 6–8 h. Schizonts were assessed for rupture by counting Giemsa-stained thin smears. The egress rate was defined as the ratio of naturally ruptured mature schizonts compared to unruptured schizonts from three replicates. For invasion assays, merozoites from egressed schizonts were allowed to invade fresh RBCs for 4–6 h, and parasitemia was determined from Giemsa-stained smears 24 h later. At least 1,000 RBCs were counted per sample in triplicate, and egress and invasion rates were calculated relative to control sgRNA (mCherry).

#### Generation of PfSET7 antibodies

The C-terminal of PfSET7 (aa 340-793) was expressed as a His-tag fusion protein in *E*. *coli* and was purified by Ni-NTA resins followed by gel filtration. Briefly, a gene fragment encoding residues 340-793 of PfSET7 was amplified using the primers 5’-GCAGCTGCATATGGAGTACGTGGAAGAGAAGCTG-3’ (forward) and 5’-CGACGGATCCTTATTCCTCAATCTG-3’ (reverse) and cloned into pET15b (Novagen) encoding an N-terminal 6×His-SUMO tag. Protein expression was induced with 0.5 mM isopropyl-D-1-thiogalactopytanoside (IPTG) at 18°C overnight in *Escherichia coli* BL-21(DE3) Cells were then resuspended in lysis buffer (20 mM HEPES pH 7.5, 100 mM NaCl, 20 mM imidazole, 10 mM beta-mercaptoethanol, 1 μg/mL DNase I) and lysed using a continuous flow cell disruptor (Constant Systems) at 25 kPSI for three passes. The target protein in the supernatant was purified by Ni-NTA affinity chromatography using a 5 mL HisTrap HP column on an ÄKTA Pure FPLC system (Cytiva) with a linear gradient of 20-600 mM imidazole. The 6×His-SUMO tag was removed by SenP2 protease (0.1-0.2 mg/mL) digestion and a second Ni-NTA affinity chromatography. To achieve higher purity, the protein was subjected to a cation-exchange chromatography on a MonoQ HR 10/10 column (Cytiva), eluting with a NaCl gradient from 100 mM to 1 M. The final purification step was size-exclusion chromatography (SEC) using a HiLoad Superdex 200 16/600 column (Cytiva). At each step of purification, protein purity was assessed by 15% SDS-PAGE. SEC fractions containing PfSET7 were pooled and concentrated to ∼10 mg/mL (Figure S6A). The purified protein was used to immunize rabbits three times to generate specific antibodies (Pocono Rabbit Farm & Laboratory Inc.) after three rabbits’ pre-immune sera were screened for non-cross-reaction with *P*. *falciparum* proteins. The rabbit anti-PfSET7 sera specifically recognized a band (94 kDa) from parasite lysates (Figure S6B).

#### PfSET7 protein expression upon dCas12a-CRISPRi

To assess the expression of SET7 in parasites before and after DiCre induction, Western blots were performed with rabbit anti-SET7 (1:50) and rabbit anti-PfAldolase antibodies as primary antibodies, followed by HRP-conjugated goat anti-rabbit IgG (Sigma) as secondary antibodies (Figure S7). Proteins were separated by SDS-PAGE, transferred to membranes and detected using the Clarity Max™ Western ECL substrate (Bio-Rad) visualized on the ChemDoc imaging system. Band densitometry was measured using ImageJ from three biological replicates.

### Quantification and statistical analysis

For all experiments, three independent biological replicates were performed. The results are presented as mean ± standard deviation (SEM) and analyzed by one- or two-way ANOVA or unpaired t-test. For RT-qPCR upon induction by rapamycin, *Δ*CTs from +Rap from 3 biological replicates were analyzed by the unpaired t-test. Statistical significance is indicated as follows: *∗p* < 0.05, *∗∗p* < 0.01, *∗∗∗p* < 0.001, and *∗∗∗∗p* < 0.0001.

## Supplemental information

Document S1. Figures S1–S9 and Table S3

Table S1. Cas9 gRNAs in 5′UTRs

Table S2. Cas12a gRNAs in 5′UTRs
