# Supplementary material for: Inducible dCas12a system expands CRISPRi/a capabilities for functional genomics in malaria parasite Plasmodium falciparum
Source: iScience. 2026 Sep 22;29(10):117704. doi: 10.1016/j.isci.2026.117704 (PMC13651822; doi:10.1016/j.isci.2026.117704)
Supplement: Supplementary file 1 [file mmc1.pdf]

iScience, Volume *29*

Supplemental information

Inducible dCas12a system expands CRISPRi/a capabilities for functional genomics in malaria parasite *Plasmodium falciparum*

Amuza Byaruhanga Lucky, XiaoLian Li, George Kobakhidze, Jose Lulo Gonzales, Chengqi Wang, Liwang Cui, Gang Dong, and Jun Miao

Figure S1. Distribution of all Cas12a sgRNAs of the Pfeba-175 gene locus. A map showing the distribution of all Cas12a sgRNAs of the 6403 bp-long *Pfeba-175* gene locus. 5' UTR is 804 bp; exons 1, 2, 3, and 4 are 4264, 79, 83, and 83 bp, respectively. A higher density of Cas12a sgRNAs is shown in the UTRs.

Figure S2. Immunoblots showing detection of dCas12-fusion proteins. Tagged dCas12-GCN5, ~180.4 kDa and dCas12-Sir2a, ~183.7 kDa in parasite lysates. Fusion proteins were detected using anti-FLAG antibody (Abcam, cat# ab205606; left panel) and anti-LbCas12a (Cpf1) antibody (Sigma-Aldrich, cat# SAB4200777; right panel). PfAldolase was used as a loading control. Anti-GFP immunoblotting was performed to confirm GFP expression and excision of the GFP cassette upon rapamycin treatment. Molecular weight markers used are a mixture of the 250 and 180 kDa protein ladders (ThermoFisher cat #26616 and 26619).

Figure S3. Growth of wild-type 3D7 and parasites expressing dCas12a-fusion proteins. Growth curves of 3D7-DiCre, dCas12a-Sir2a, and dCas12a-GCN5 parasite backgrounds before gRNA plasmids were transfected into them. Rapamycin induction did not affect parasite growth.

Figure S4. CRISPRi/a negative control sgRNAs. Selection of CRISPRi/a negative control gRNAs. **A**. Two designed decoy sgRNAs targeting the 5' UTR of *Pfcg6* and the coding region of *mCherry*. **B**. Growth curves of dCas12a-Sir2a or -GCN5 parasite lines containing sgRNA targeting *Pfcg6* and *mCherry*. The arrows and wavy lines indicate major TSSs and sgRNA locations, with their orientations relative to the start codon.

Figure S5. sgRNA folding energy compared with activity. The predicted minimum free energy (ΔG, kcal/mol) values for each guide RNA were plotted against mean fold change (FC) for gene repression or activation efficiency. More negative ΔG indicates stronger predicted RNA folding stability. Each point represents an individual guide RNA: blue =gRNA1, green =gRNA2, gray =gRNA3. mCherry gRNA served as a control. Linear regression analysis shows no strong correlation between predicted RNA folding stability and guide performance ($R^2 = 0.12$ for repression and $R^2 = 0.11$ for activation), suggesting that variation in gRNA performance is less likely to be determined by RNA secondary structure.

Figure S6. A. **Recombinant PfSET7 protein used for antibody generation. A.** The purified C-terminal region of PfSET7 (residues 340-793) containing the conserved functional domains was purified by Ni-NTA resins followed by gel filtration and concentrated to ~10 mg/ml. The yield and purity were analyzed by 15% SDS-PAGE. A prominent band corresponding to the expected molecular mass of ~54 kDa was observed. **B.** Western blots showing the detection of a specific band against PfSET7 in 3D7 lysate. No signal was observed in the blot probed with pre-immune serum.

Figure S7. Immunoblots showing CRISPRi of PfSET7 protein levels. Western blots showing detection of PfSET7 (94 kDa) and PfAldolase (41 kDa) in parasite lysates. Blots are representative of three independent biological replicates. PfAldolase was used as a loading control. The molecular weight markers used are a mixture of the 250- and 180-kDa protein ladders (Thermo Fisher Cat #26616 and 26619).

Figure S8. Pfset7 RT-qPCR transcript levels in dCas12a-GCN5 parasites A. RT-qPCR analysis of Pfset7 transcript levels in dCas12a-GCN5 parasites with individual sgRNAs. **B**. Growth curves in dCas12a-GCN5 parasites expressing Pfset7 sgRNAs or controls following Rap treatment (ANOVA, $P<0.0001$). Data in A represent mean ± SEM from three biological replicates; Numbers in bars indicate fold-change (FC) values (t-tests: *, **, ****: P<0.05, 0.01, 0.0001, respectively).

Figure S9. Plasmid maps of vectors expressing dCas12 fusions. Plasmid maps showing the final expression vectors (**A**) pUF-dCas12-BSD-GCN5 and **(B)** pUF-dCas12-BSD-Sir2a

Tables S3. **Primer information.** gRNA, primer, and plasmid information

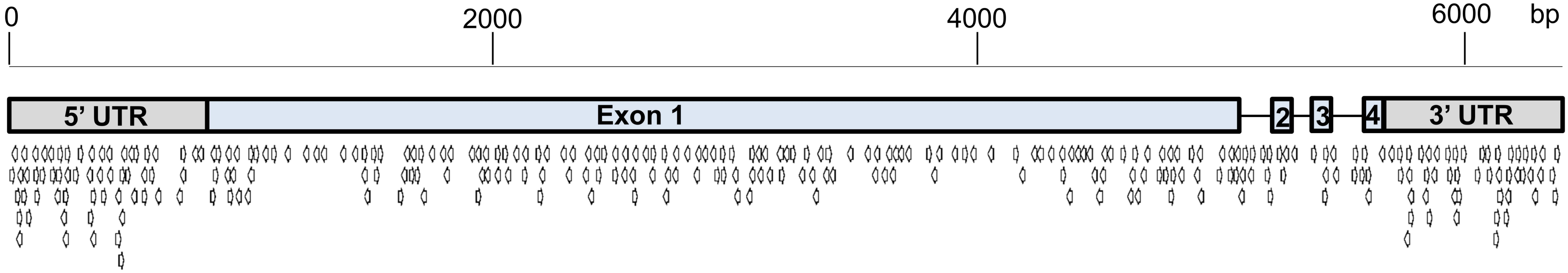

Figure S1. Distribution of all Cas12a sgRNAs of the Pfeba-175 gene locus. A map showing the distribution of all Cas12a sgRNAs of the 6403 bp-long *Pfeba-175* gene locus. 5' UTR is 804 bp; exons 1, 2, 3, and 4 are 4264, 79, 83, and 83 bp, respectively. A higher density of Cas12a sgRNAs is shown in the UTRs.

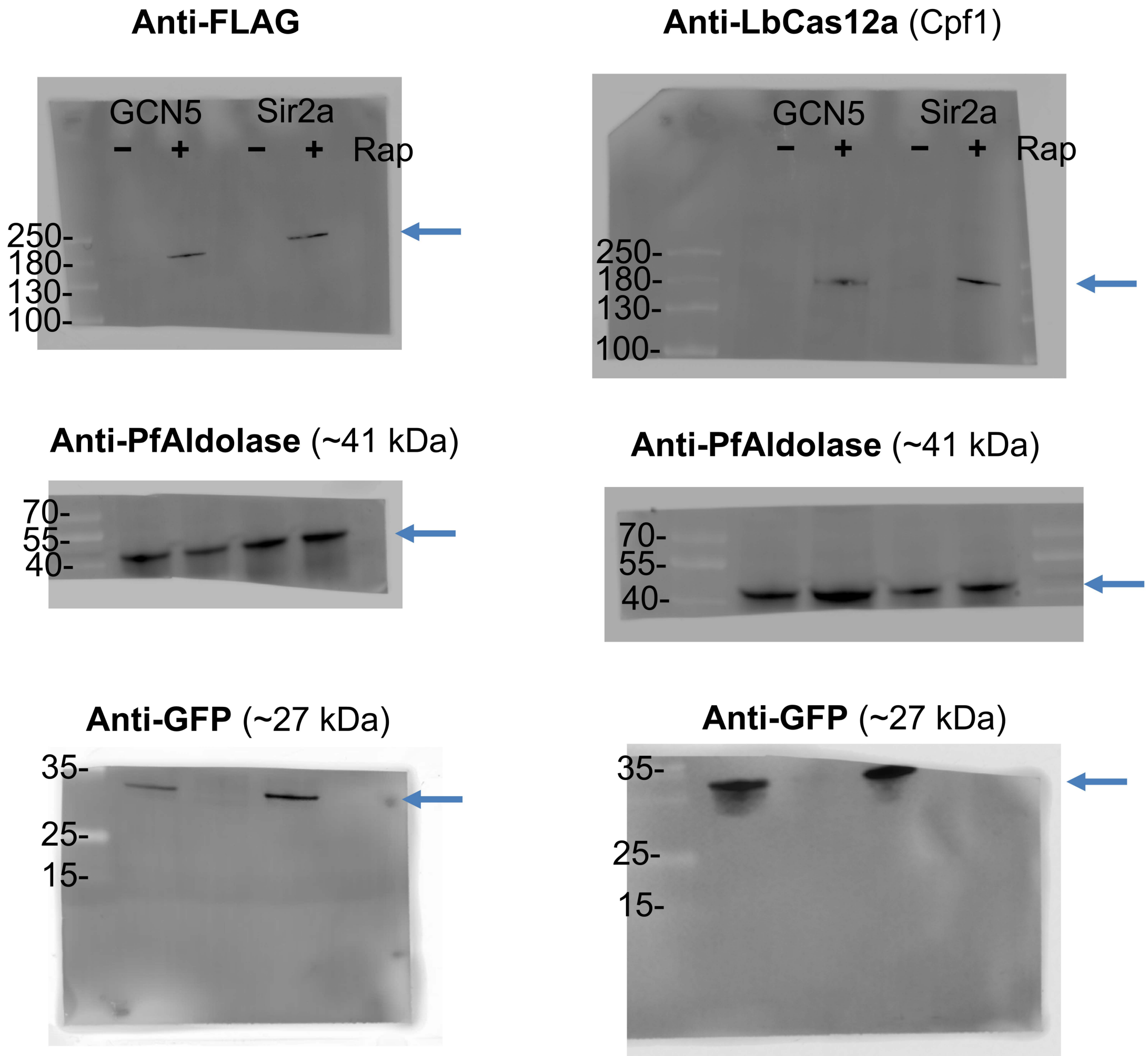

Figure S2. Immunoblots showing detection of dCas12-fusion proteins. Tagged dCas12-GCN5, ~180.4 kDa and dCas12-Sir2a, ~183.7 kDa in parasite lysates. Fusion proteins were detected using anti-FLAG antibody (Abcam, cat# ab205606; left panel) and anti-LbCas12a (Cpf1) antibody (Sigma-Aldrich, cat# SAB4200777; right panel). PfAldolase was used as a loading control. Anti-GFP immunoblotting was performed to confirm GFP expression and excision of the GFP cassette upon rapamycin treatment. Molecular weight markers used are a mixture of the 250 and 180 kDa protein ladders (ThermoFisher cat #26616 and 26619).

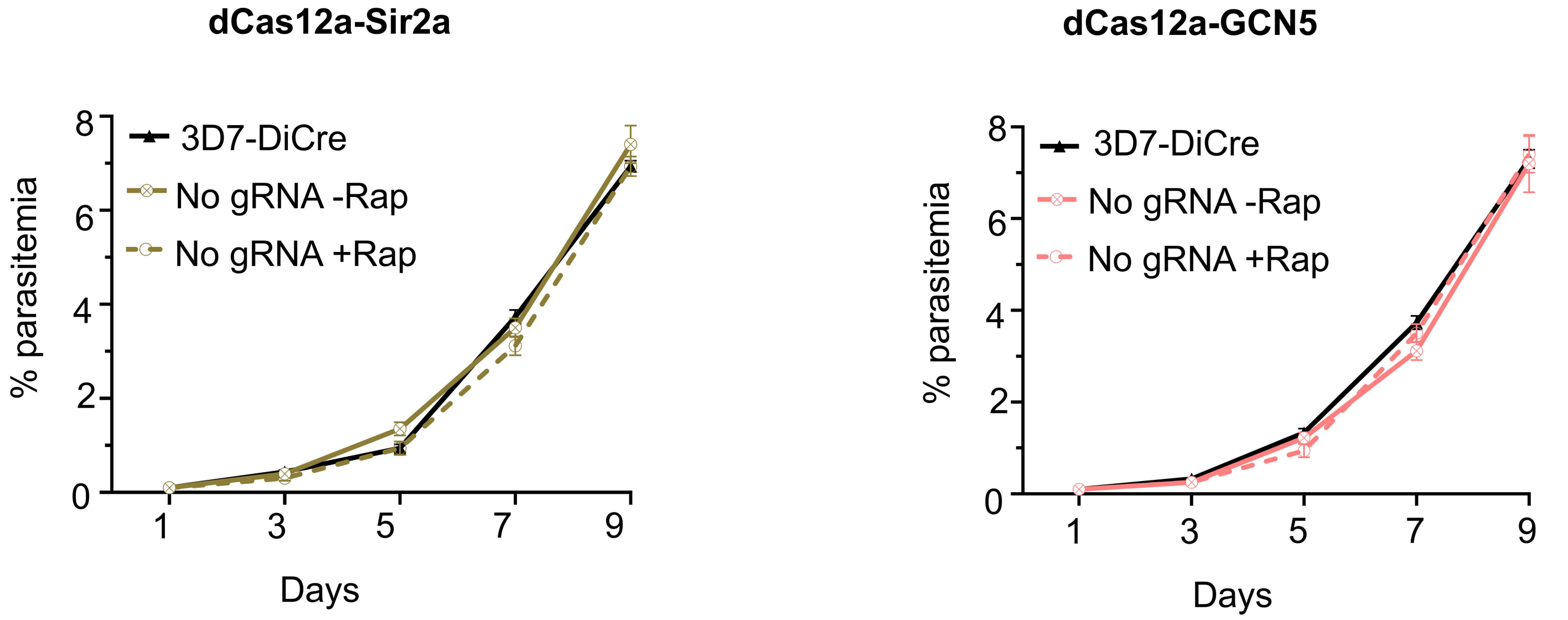

Figure S3. Growth of wild-type 3D7 and parasites expressing dCas12a-fusion proteins. Growth curves of 3D7-DiCre, dCas12a-Sir2a, and dCas12a-GCN5 parasite backgrounds before gRNA plasmids were transfected into them. Rapamycin induction did not affect parasite growth.

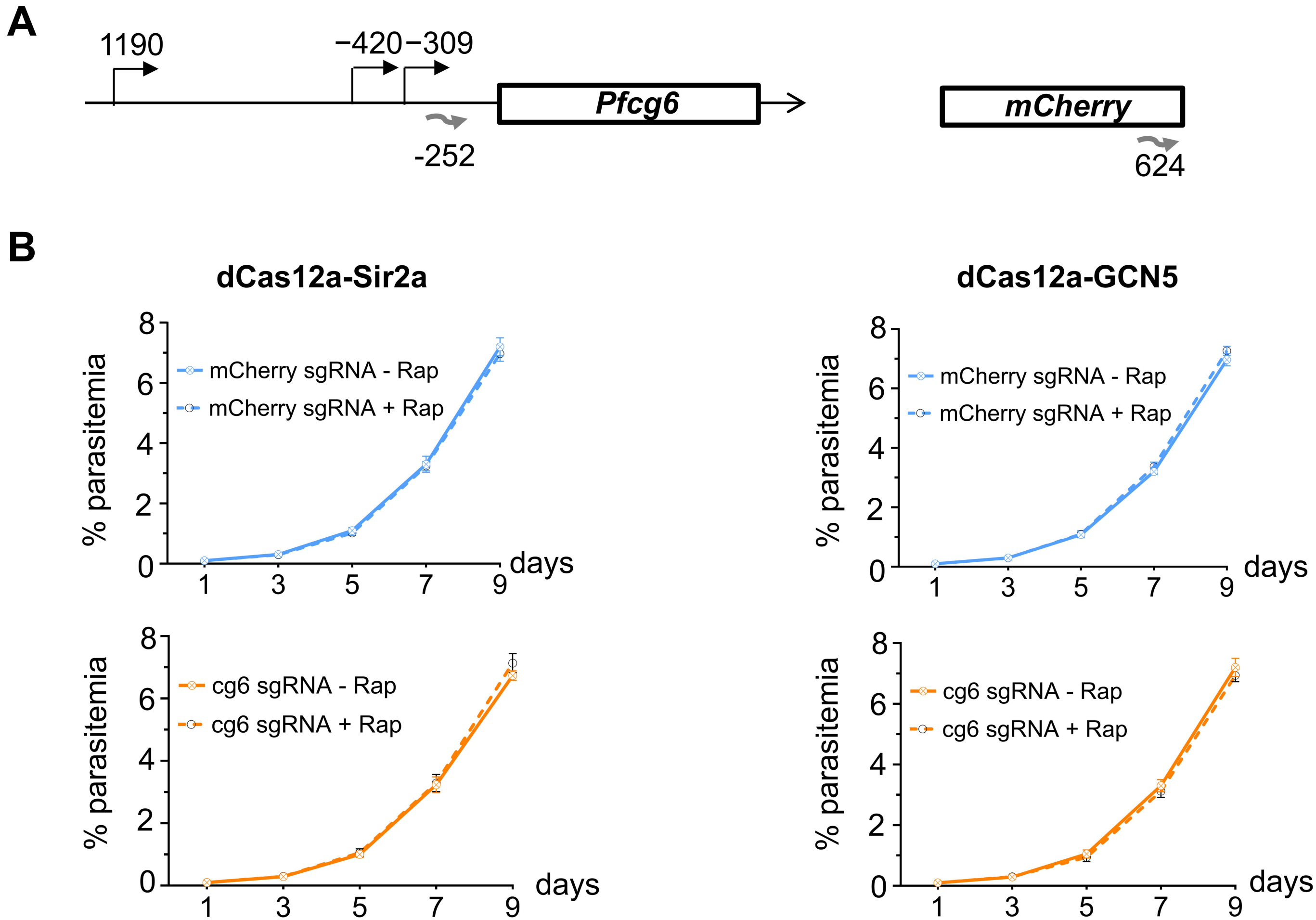

Figure S4. CRISPRi/a negative control sgRNAs. Selection of CRISPRi/a negative control gRNAs. **A**. Two designed decoy sgRNAs targeting the 5' UTR of *Pfcg6* and the coding region of *mCherry*. **B**. Growth curves of dCas12a-Sir2a or -GCN5 parasite lines containing sgRNA targeting *Pfcg6* and *mCherry*. The arrows and wavy lines indicate major TSSs and sgRNA locations, with their orientations relative to the start codon.

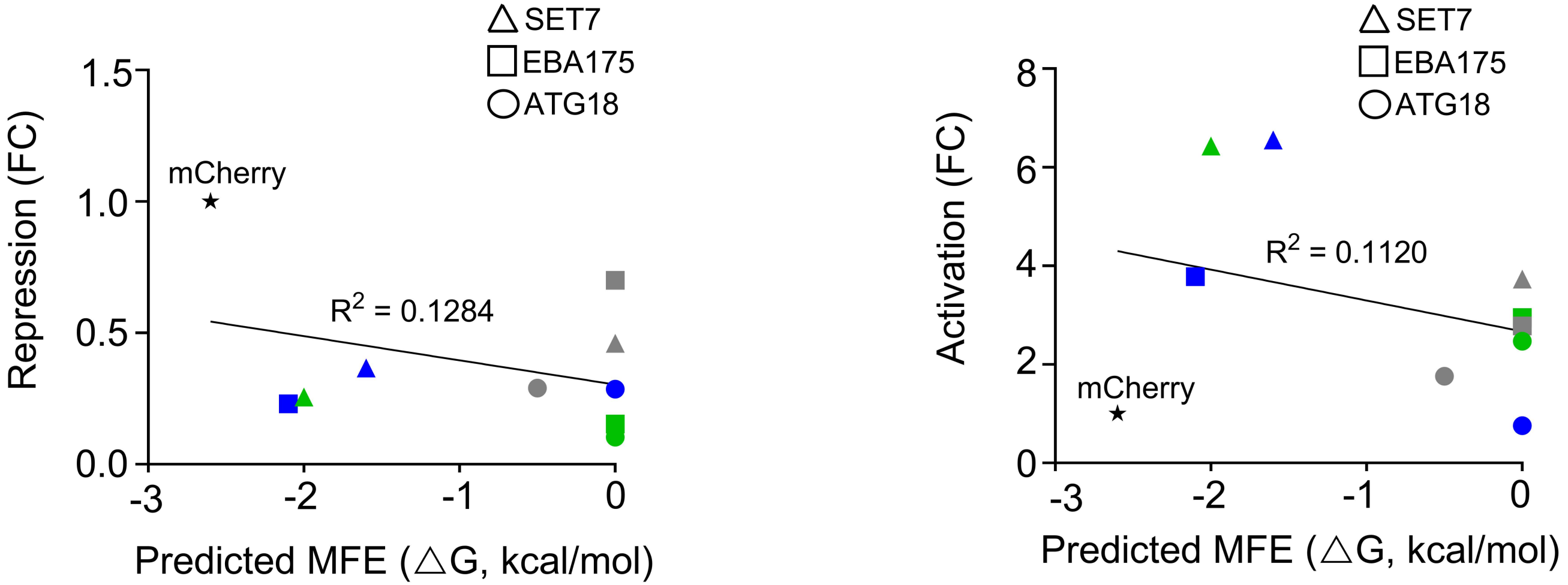

Figure S5. sgRNA folding energy compared with activity. The predicted minimum free energy (ΔG, kcal/mol) values for each guide RNA were plotted against mean fold change (FC) for gene repression or activation efficiency. More negative ΔG indicates stronger predicted RNA folding stability. Each point represents an individual guide RNA: blue =gRNA1, green =gRNA2, gray =gRNA3. mCherry gRNA served as a control. Linear regression analysis shows no strong correlation between predicted RNA folding stability and guide performance (R^2 = 0.12 for repression and R^2 = 0.11 for activation), suggesting that variation in gRNA performance is less likely to be determined by RNA secondary structure.

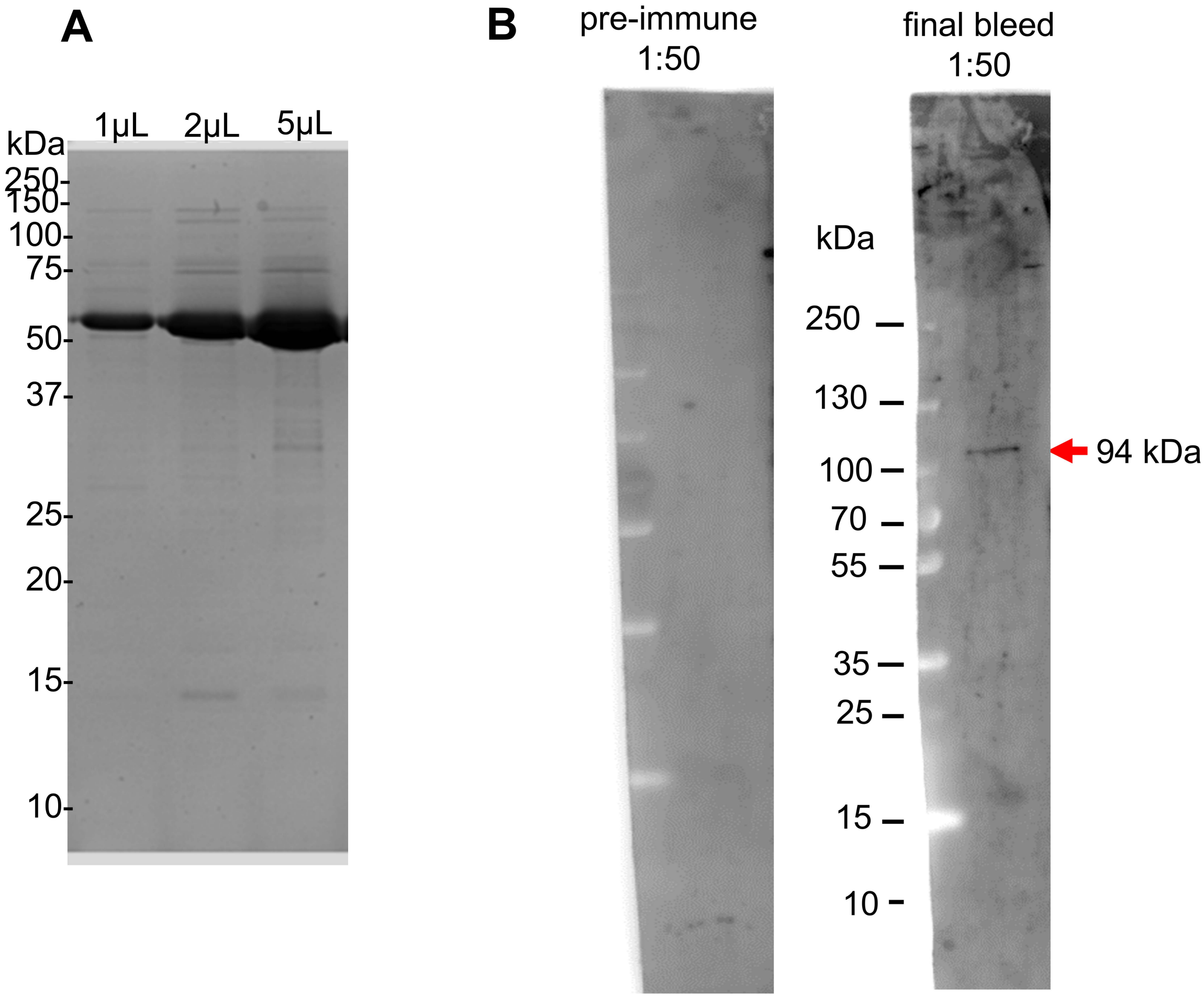

Figure S6. A. **Recombinant PfSET7 protein used for antibody generation. A.** The purified C-terminal region of PfSET7 (residues 340-793) containing the conserved functional domains was purified by Ni-NTA resins followed by gel filtration and concentrated to ~10 mg/ml. The yield and purity were analyzed by 15% SDS-PAGE. A prominent band corresponding to the expected molecular mass of ~54 kDa was observed. **B.** Western blots showing the detection of a specific band against PfSET7 in 3D7 lysate. No signal was observed in the blot probed with pre-immune serum.

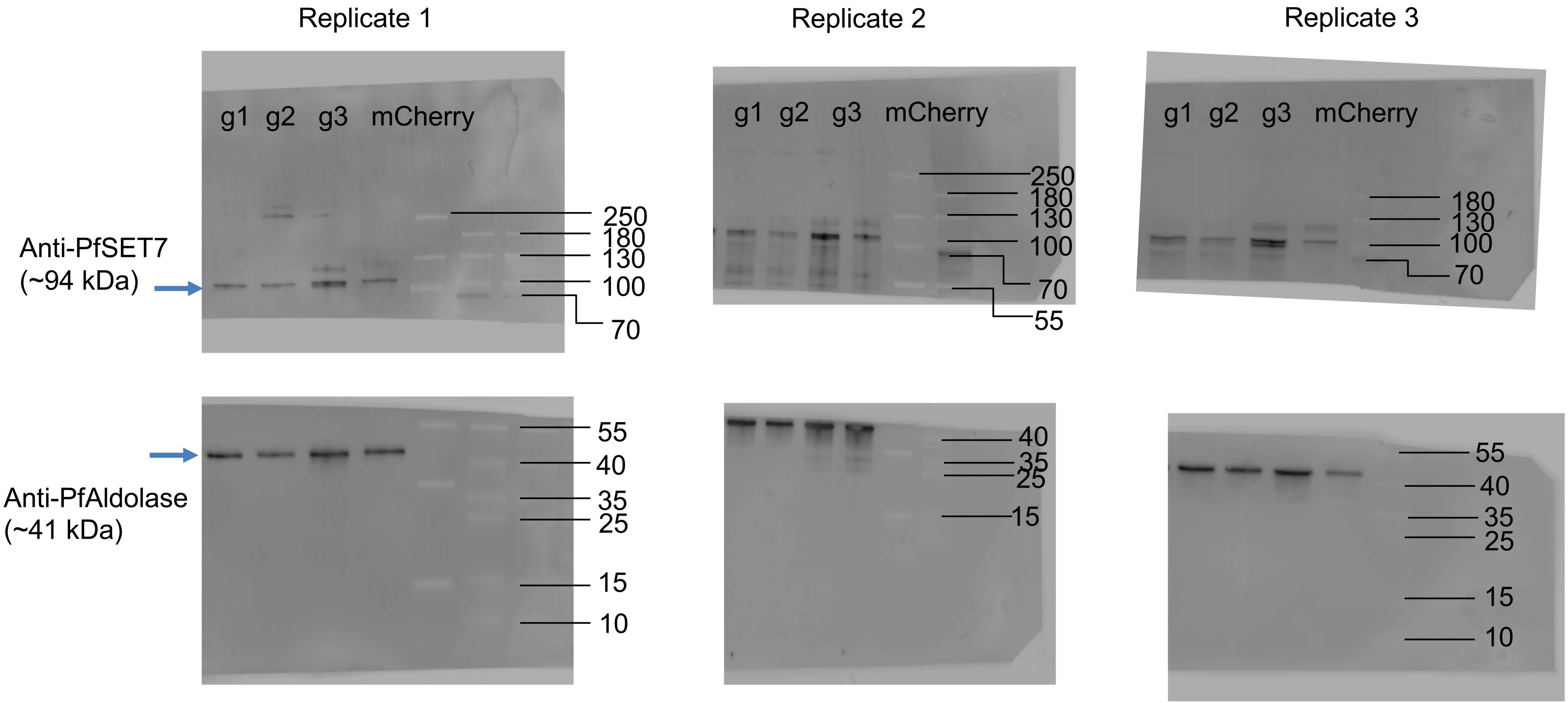

Figure S7. Immunoblots showing CRISPRi of PfSET7 protein levels. Western blots showing detection of PfSET7 (94 kDa) and PfAldolase (41 kDa) in parasite lysates. Blots are representative of three independent biological replicates. PfAldolase was used as a loading control. The molecular weight markers used are a mixture of the 250- and 180-kDa protein ladders (Thermo Fisher Cat #26616 and 26619).

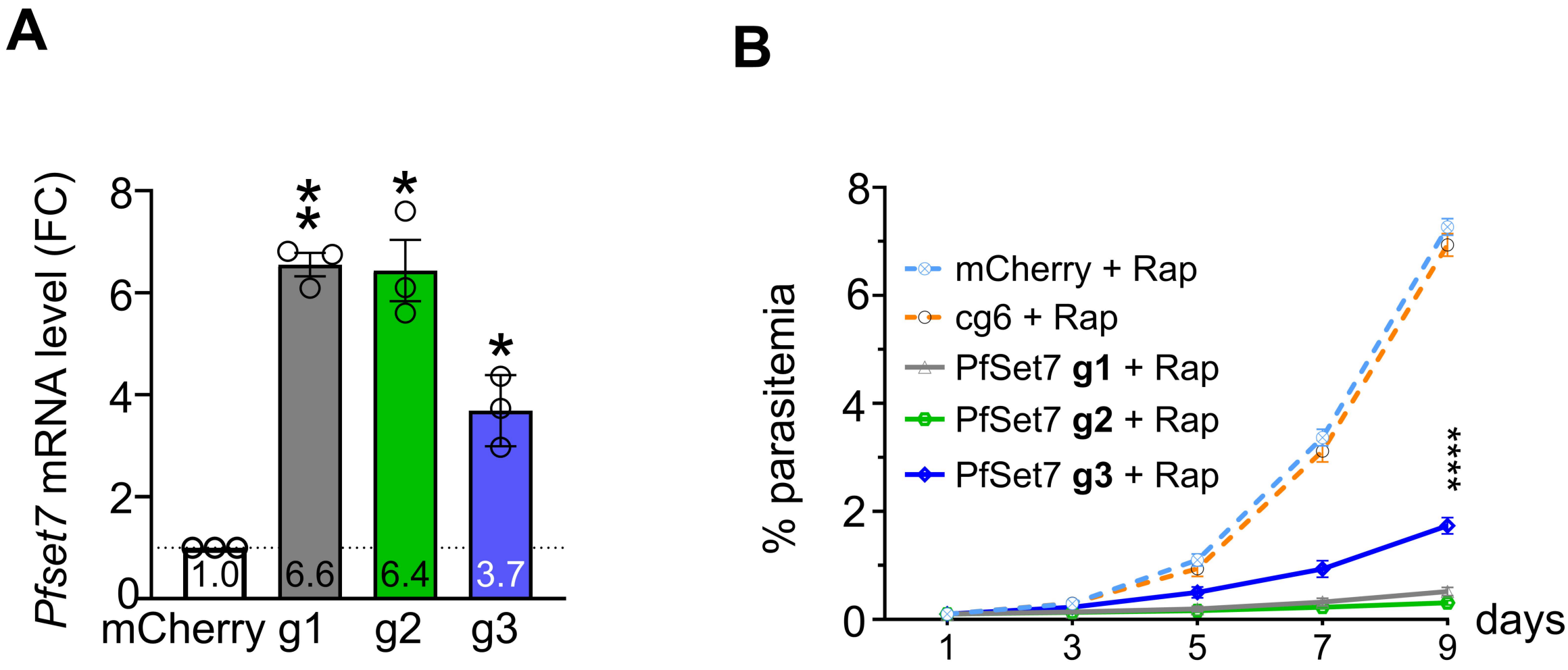

Figure S8. Pfset7 RT-qPCR transcript levels in dCas12a-GCN5 parasites
A. RT-qPCR analysis of Pfset7 transcript levels in dCas12a-GCN5 parasites with individual sgRNAs. **B**. Growth curves in dCas12a-GCN5 parasites expressing Pfset7 sgRNAs or controls following Rap treatment (ANOVA, *P<0.0001*). Data in A represent mean ± SEM from three biological replicates; Numbers in bars indicate fold-change (FC) values (t-tests: *, **, **** : *P*<0.05, 0.01, 0.0001, respectively).

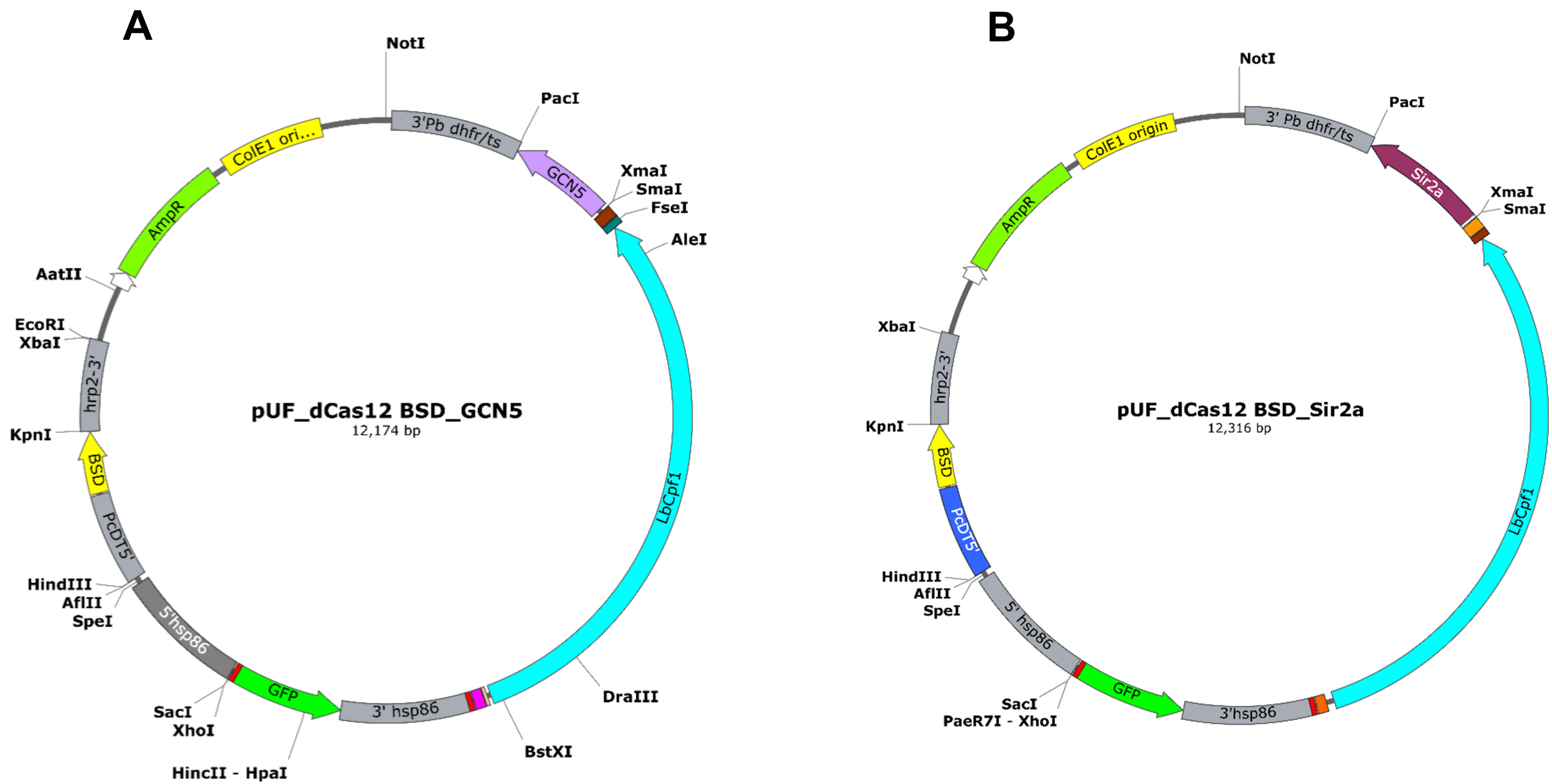

Figure S9. Plasmid maps of vectors expressing dCas12 fusions. Plasmid maps showing the final expression vectors (**A**) pUF-dCas12-BSD-GCN5 and **(B)** pUF-dCas12-BSD-Sir2a

Primer name	5'-3' Sequence
**Primers for plasmid construction**	
dcas12.inf_F	ATTAAGCTGCCATATCCTTAATTAACCCGGGATAGTCGGGGAC
dcas12.inf_R	CCCAGCAGCCATGAGCAAGCTGGAGAAGTTTAC
HAT.infF	GCTGCCATATCCTTAATTAATATAAAATGTATAGCTTTCTTTACT
HAT.infR	GTCCCCGACTATCCCGGGGGCTCTGGCAGCGGCAGCAT
HDAC.infF	GCTGCCATATCCTTAATTAATTTCACTTGACCCTTTTAATATATT
HDAC.infR	GTCCCCGACTATCCCGGGGGCTCTGGCAGCGGCAGC
**sgRNA primers**	
EBA175 sgRNA1-F	tcttgtagatcttaaTATCTTAGTTTCTATGATATAAAttaagtttttatta
EBA175 sgRNA1-R	taataaaaaacttaaTTTATATCATAGAAACTAAGATAttaagatctacaaga
EBA175 sgRNA2-F	tcttgtagatcttaaTGGTGCCAGATTATAGAACAATAttaagtttttatta
EBA175 sgRNA2-R	taataaaaaacttaaTATTGTTCTATAATCTGGCACCAttaagatctacaaga
EBA175 sgRNA3-F	tcttgtagatcttaaCACTATTAAGAAAAAAAAATTTAttaagtttttatta
EBA175 sgRNA3-R	taataaaaaacttaaTAAATTTTTTTTTCTTAATAGTGttaagatctacaaga
ATG18 sgRNA1-F	tcttgtagatcttaaCCACATTCATTATAGAAGAAATTAttaagtttttatta
ATG18 sgRNA1-R	taataaaaaacttaaTAATTTCTTCTATAATGAATGTGGttaagatctacaaga
ATG18 sgRNA2-F	tcttgtagatcttaaTGAAAATATGAAAAAGATATACTttaagtttttatta
ATG18 sgRNA2-R	taataaaaaacttaaAGTATATCTTTTTCATATTTTCAttaagatctacaaga
ATG18 sgRNA3-F	tcttgtagatcttaaAAGGGTAAGGTATATTATTAAATttaagtttttatta
ATG18 sgRNA3-R	taataaaaaacttaaATTTAATAATATACCTTACCCTTttaagatctacaaga
SET7 sgRNA1-F	tcttgtagatcttaaGCTTGATATGAATTGTTAAATTTttaagtttttatta
SET7 sgRNA1-R	taataaaaaacttaaAAATTTAACAATTCATATCAAGCttaagatctacaaga
SET7 sgRNA2-F	tcttgtagatcttaaTGTGTCAAAATATGTATACACACttaagtttttatta
SET7 sgRNA2-R	taataaaaaacttaaGTGTGTATACATATTTTGACACAttaagatctacaaga
SET7 sgRNA3-F	tcttgtagatcttaaTATTATGAGAAAAATAAACATTCttaagtttttatta
SET7 sgRNA3-R	taataaaaaacttaaGAATGTTTATTTTTCTCATAATAttaagatctacaaga
**Decoy sgRNA primers**	
mCherry sgRNA1-F	tcttgtagatcttaaCCACAACGAGGACTACACCATCGttaagtttttatta
mCherry sgRNA1-R	taataaaaaacttaaCGATGGTGTAGTCCTCGTTGTGGttaagatctacaaga
cg6 sgRNA1-F	tcttgtagatcttaaTTCATCCTTATAGTGGCATATATttaagtttttatta
cg6 sgRNA1-R	taataaaaaacttaaATATATGCCACTATAAGGATGAAttaagatctacaaga
**gRNA array primers**	
EBA175 array-F	tcttgtagatcttaaTATCTTAGTTTCTATGATATAAAaatttctactcttgtagatTGGTGCCAGATTATAGAACAATAaatttctactcttgtagatCACTATTAAGAAAAAAAAATTTAtaatttctactcttgtagatttaagtttttatta
EBA175 array-R	taataaaaaacttaaatctacaagagtagaaattaTAAATTTTTTTTTCTTAATAGTGatctacaagagtagaaattTATTGTTCTATAATCTGGCACCAatctacaagagtagaaattTTTATATCATAGAAACTAAGATAttaagatctacaaga
ATG18 array-F	tcttgtagatcttaaCCACATTCATTATAGAAGAAATTAtaatttctactcttgtagatTGAAAATATGAAAAAGATATACTtaatttctactcttgtagatAAGGGTAAGGTATATTATTAAATtaatttctactcttgtagatttaagtttttatta
ATG18 array-R	taataaaaaacttaaatctacaagagtagaaattaATTTAATAATATACCTTACCCTTatctacaagagtagaaattaAGTATATCTTTTTCATATTTTCAatctacaagagtagaaattaTAATTTCTTCTATAATGAATGTGGttaagatctacaaga
SET7 array-F	tcttgtagatcttaaGCTTGATATGAATTGTTAAATTTtaatttctactcttgtagatTGTGTCAAAATATGTATACACACtaatttctactcttgtagatTATTATGAGAAAAATAAACATTCtaatttctactcttgtagatttaagtttttatta
SET7 array-R	taataaaaaacttaaatctacaagagtagaaattaGAATGTTTATTTTTCTCATAATAatctacaagagtagaaattaGTGTGTATACATATTTTGACACAatctacaagagtagaaattaAAATTTAACAATTCATATCAAGCttaagatctacaaga
**Primers for qPCR**	
EBA175-F	GGAAGAGTTATGGAACTCCAG
EBA175-R	GTAATAGTAACGATGCGATGTC
ATG18-F	CCAGGATTATGGTTGTTTGTG
ATG18-R	GTTAGTTTCGCTATTTCCCG
SET7-F	GAGTGAATACCATGATGA
SET7-R	AGCTAAGGCATCAGACA
seryl-tRNA synthetase F	AAGTAGCAGGTCATCGTGGTT
seryl-tRNA synthetase R	TTCGGCACATTCTTCCATAA
